# Cell Death Is Not Sufficient for the Restriction of Potato Virus Y Spread in Hypersensitive Response-Conferred Resistance in Potato

**DOI:** 10.3389/fpls.2018.00168

**Published:** 2018-02-15

**Authors:** Tjaša Lukan, Špela Baebler, Maruša Pompe-Novak, Katja Guček, Maja Zagorščak, Anna Coll, Kristina Gruden

**Affiliations:** ^1^Department of Biotechnology and Systems Biology, National Institute of Biology, Ljubljana, Slovenia; ^2^Jožef Stefan International Postgraduate School, Ljubljana, Slovenia

**Keywords:** *Solanum tuberosum* L., potato virus Y, *Potyviridae*, hypersensitive response, programmed cell death, necrotic lesion, callose deposits, salicylic acid

## Abstract

Hypersensitive response (HR)-conferred resistance to viral infection restricts the virus spread and is accompanied by the induction of cell death, manifested as the formation of necrotic lesions. While it is known that salicylic acid is the key component in the orchestration of the events restricting viral spread in HR, the exact function of the cell death in resistance is still unknown. We show that potato virus Y (PVY) can be detected outside the cell death zone in *Ny-1*-mediated HR in potato plants (cv. Rywal), observed as individual infected cells or small clusters of infected cells outside the cell death zone. By exploiting the features of temperature dependent *Ny-1*-mediated resistance, we confirmed that the cells at the border of the cell death zone are alive and harbor viable PVY that is able to reinitiate infection. To get additional insights into this phenomenon we further studied the dynamics of both cell death zone expansion and occurrence of viral infected cell islands outside it. We compared the response of Rywal plants to their transgenic counterparts, impaired in SA accumulation (NahG-Rywal), where the lesions occur but the spread of the virus is not restricted. We show that the virus is detected outside the cell death zone in all lesion developmental stages of HR lesions. We also measured the dynamics of lesions expansion in both genotypes. We show that while rapid lesion expansion is observed in SA-depleted plants, virus spread is even faster. On the other hand the majority of analyzed lesions slowly expand also in HR-conferred resistance opening the possibility that the infected cells are eventually engulfed by cell death zone. Taken altogether, we suggest that the HR cell death is separated from the resistance mechanisms which lead to PVY restriction in *Ny-1* genetic background. We propose that HR should be regarded as a process where the dynamics of events is crucial for effectiveness of viral arrest albeit the exact mechanism conferring this resistance remains unknown.

## Introduction

Hypersensitive response (HR)-conferred resistance is an effective defense response determined by the resistance (R) genes and is manifested as the formation of necrotic lesions (Pontier et al., 1998). Our understanding of the mechanisms that underlie HR-conferred resistance against pathogens is still incomplete. Following pathogen recognition by the R protein, transduction of the perceived signal to the inducers of cell death is engaged, programmed cell death (PCD) is induced and the pathogen is restricted to the site of inoculation (Singh and Upadhyay, 2013). In plants, we distinguish two main types of PCD, vacuolar and necrotic. The PCD observed in the HR combines the signs of both types and should be therefore considered as a separate type of PCD (Fomicheva et al., 2012). The question if the programmed cell death of a limited region of cells restricts pathogen proliferation or if lesion formation is merely collateral damage of the resistance mechanisms has long been discussed (Richael and Gilchrist, 1999). To this end, several mutants have been produced to study the role of HR cell death in the restriction of the pathogen growth (Morel and Dangl, 1997; Greenberg and Yao, 2004). What has emerged is that the role of cell death in resistance depends on the type of host–pathogen interaction. In general, it is more likely that cell death contributes to pathogen arrest of obligate pathogens that require living cells in order to replicate (Greenberg and Yao, 2004). For example, the cell death was envisioned as directly responsible for the restriction of oomycete multiplication (Hein et al., 2009; Gilroy et al., 2011). On the other hand, several examples in which the restriction of the (hemi) biotrophic pathogens spread was shown to be uncoupled from the HR cell death were reported (reviewed in Künstler et al., 2016). The attenuation of the HR cell death by modifying *Arabidopsis* type I metacaspase regulatory module (Coll et al., 2010) did not lead to enhanced biotrophic oomycete *Hyaloperonospora arabidopsidis* and hemibiotrophic bacteria *Pseudomonas syringae* proliferation. In addition, results obtained by the overexpression of the R-gene interactor RIN13 (Al-Daoude et al., 2005) or by the mutation of a cyclic nucleotide-gated ion channel DND1 (Yu et al., 1998; Clough et al., 2000) further confirmed that restriction of *P. syringae* growth could occur in the absence of HR cell death. Also in plant-virus interaction, NbMAPKKKα silencing (Komatsu et al., 2010) or introgression of *CCD1* from *Nicotiana bigelovii* into *Nicotiana clevelandii* (Cawly et al., 2005) suppressed cell death but did not lead to enhanced plantago asiatica mosaic virus (PlAMV), potato virus X (PVX) and cauliflower mosaic virus (CaMV) proliferation. Similarly, treatment with reactive oxygen species or antioxidant components resulted in the suppression of the HR cell death, while tobacco mosaic virus (TMV) spread was not restricted (Farkas et al., 1960; Király et al., 2008; Hafez et al., 2012).

In addition to the direct evidence mentioned above, indirect data also suggest that HR cell death can be dispensable for pathogen restriction in some pathosystems. The mutants with unaltered HR-like phenotype, but with an inability to restrict the pathogen growth confirm the theory that the HR cell death may not be sufficient for pathogen arrest when other defenses are compromised (Greenberg and Yao, 2004). For example, the resistance against turnip crinkle virus (TCV) was compromised in *eds5, pad4*, and *sid2* mutants without affecting HR cell death (Chandra-Shekara et al., 2004), and the disabled interaction between the WRKY70 transcription factor and the RCY1 resistance protein disactivated cucumber mosaic virus (CMV) restriction of movement with no effect on HR cell death (Ando et al., 2014).

*Potato virus Y* (PVY), a member of the family *Potyviridae*, is currently considered as the most economically harmful virus affecting cultivated potatoes (Karasev and Gray, 2013). In potato cv. Rywal, HR is mediated by *Ny-1* resistance gene and is manifested as the formation of necrotic lesions on inoculated leaves 3 days post inoculation (dpi) (Szajko et al., 2008). While it is known that salicylic acid (SA) is the key component in the orchestration of the events restricting virus spread in HR (Baebler et al., 2014), the exact function of the cell death process in resistance is still unknown. The aim of the present study was to investigate the relation between cell death and restriction of PVY spread in *Ny-1* genetic background. Therefore, we studied the cells in the lesions and surrounding the lesions formed after PVY infection in potato cv. Rywal in comparison to its transgenic counterpart impaired in SA accumulation (NahG-Rywal), where the lesions occur but the spread of the virus is not restricted. In this genotype we observed concentric viral spread, similarly as observed in susceptible tobacco leaves (Rupar et al., 2015). Interestingly however, we observed individual virus-infected cells or small clusters of infected cells outside of the cell death zone also in cv. Rywal. We confirmed that the infected cells at the periphery of the cell death zone are alive and able to reinitiate infection. To get additional insights into this phenomenom we further studied the dynamics of both cell death zone expansion and occurrence of viral infected cell islands outside it.

## Methods

### Plant material and virus inoculation

Potato (*Solanum tuberosum* L.) cv. Rywal plants and plants expressing NahG transgene (NahG-Rywal; Baebler et al., 2014) were obtained from the Institute of Plant Breeding and acclimatization—National Research Institute, Młochów (Poland). Plants were grown in stem node tissue culture. Two weeks after node segmentation, they were transferred to soil in a growth chamber and grown for 4 weeks under controlled environmental conditions as previously described (Baebler et al., 2011) unless otherwise stated. Green fluorescent protein-tagged PVY N605 infective clone (PVY N605-GFP) was introduced in tobacco plants by Rupar et al. using biolistic bombardment (Rupar et al., 2015). We obtained frozen inoculum containing leaf extracts of inoculated *Nicotiana tabacum* L. var Xanthi nc. leaves after several passages of infection. The virus was further multiplied and maintained in tobacco plants, *N. tabacum* L. var Xanthi nc. We ensured that the strength of the inoculum used for the inoculation of potato plants was comparable among all experiments. First, we used aliquots of frozen inoculum from the same batch to inoculate tobacco plants in each of the related experiments. After the virus had spread to tobacco systemic leaves, we confirmed its presence under confocal microscope. Next, only the infected areas of the leaves with comparable intensity of GFP fluorescence were used for inoculum preparation. Selected areas of infected tobacco leaves were weighed and ground in 20 mM phosphate buffer supplemented with DIECA in the ratio 1:4 prior potato inoculation in all experiments. PVY inoculation was performed as described elsewhere (Baebler et al., 2009, 2011). In short, first three bottom fully developed leaves of 4 weeks old plants were dusted with carborundum powder and rubbed with the inoculum (~60 μl per leaf). After 10 min, the leaves were washed with tap water.

### Confocal and light microscopy

Green fluorescent protein (GFP) and Aniline blue fluorochrome fluorescence were followed on the upper side of the leaf using confocal microscope (Leica TCS LSI macroscope with Plan APO 5 × objective, Leica Microsystems). The 488 nm laser was used for the excitation of GFP and background chloroplast fluorescence and the 405 nm laser was used for the excitation of Aniline blue fluorochrome. The GFP emission was measured in the window from 505 to 530 nm, the background chloroplast fluorescence emission was measured in the window from 690 to 750 nm and the Aniline blue fluorochrome emission was measured in the window from 460 to 500 nm. Fluorescence emissions were collected sequentially through two or three channels. Regions of interest were scanned unidirectionally with frame average at least 2 and scan speed 400 Hz. The images were processed and merged using Leica LAS X software (Leica Microsystems). Images are presented as maximum projections from Z-stacks or single sections. Z-stack was sized to cover the whole epidermis (~30 μm) and at least 20 μm of the mesophyll.

### Tracking of PVY N605-GFP on detached leaves

To follow virus spread around the cell death zone and to count the number of lesions with the virus outside cell death zone, one or two Rywal and NahG-Rywal plants were analyzed at eight discrete time points from 3 to 12 dpi, one leaf per plant, one to ten lesions per leaf and two regions of interest per lesion (Supplementary Table 1). Leaf was detached from the plant on the particular day post inoculation (dpi) and analyzed by confocal microscope. Lesions were scanned in the channel for the background chloroplast fluorescence detection to estimate the location of the lesion. The edge between the cell death zone and normal tissue was determined using brightfield. We confirmed the virus presence indirectly by the PVY N605-GFP accumulation. The cell was considered to contain the PVY N605-GFP only when the GFP fluorescence was observed in the cytoplasm. In addition, lambda-scan was performed to differentiate between GFP spectra and the spectra of the background signal. The spectrum was attributed to GFP fluorescence by comparing it with publicly available spectra of GFP. We define the lesions in which we detected PVY outside death zone as positive lesions. As a negative control, plants were infected with PVY without GFP tag. We merged the results of seven independent experiments to calculate the percentage of positive lesions in each plant/time point as the number of lesions in which the PVY N605-GFP accumulation was detected in at least one cell outside the cell death zone, divided by the number of all analyzed lesions.

To follow virus spread around the lesions in cv. Rywal at higher temperature, PVY N605-GFP inoculated plants were kept at 22°C until the lesions were formed. The inoculated leaf was detached from cv. Rywal at 4 dpi, cut into pieces and placed on 1.5% (w/v) agar plate and kept at 22°C. At four consecutive days (4, 5, 6, and 7 dpi) the plate was transferred to 28°C for 2 h. The GFP fluorescence intensity of the same area of interest was followed after temperature shift using confocal microscope. The experiment was repeated twice.

### Dynamics of lesion expansion

To follow the speed of the lesion expansion, plants from 13 independent experiments for cv. Rywal (more than 100 lesions) and five independent experiments for NahG-Rywal (48 lesions) were continuously monitored using Dino-Lite Edge AM7915MZTL digital microscope (AnMo Electronics Corporation) from immediately after the inoculation on or only at later time points.

### Dynamics of lesion formation

16 Rywal and 11 NahG-Rywal plants were inoculated as described above. Lesions were counted on each inoculated leaf at 3, 4, 5, 6, 7, 9, and 11 dpi. Any discrepancy in lesion number counted by eye (Figure S3, marked with green) or under confocal microscope (Supplementary Table 1, exp2) at early time points is due to the lesions size, as some lesions were not visible by eye. Statistical analysis was conducted separately per cultivar using R3.4.0 (R Core Team, 2017). Samples (leaves) with more than 50% missing values and maximal number of lesions lower than 4 were filtered out. Cumulative sums of lesion counts at consecutive time points were used to compute the cumulative relative frequency distribution (CRFD) per sample. The filtered data set was imputed using cumulative maximum, and only samples with monotonically non-decreasing functions were kept. Logistic function, cumulative distribution function (CDF) of the logistic distributions, is a solution for a population growth with a carrying capacity model. Nonlinear regression model with the self-starting logistic growth function approach was used to get the function that best describes the data. CDFs were used to predict a response and calculate the probability of lesion forming before or between measured time points.

### Callose staining

The leaves of two cv. Rywal plants were inoculated as described above. Aniline blue fluorochrome solution was prepared according to the provider's instructions (Biosupplies Australia) and infiltrated with a syringe into the second fully developed inoculated leaf at 4 and 6 dpi. The GFP and Aniline blue fluorochrome fluorescence were followed inside and outside the cell death zone on detached leaves using confocal microscope. The experiment was repeated twice.

## Results

### PVY is multiplying outside the necrotic lesions in HR-conferred resistance

**Figure 1:**
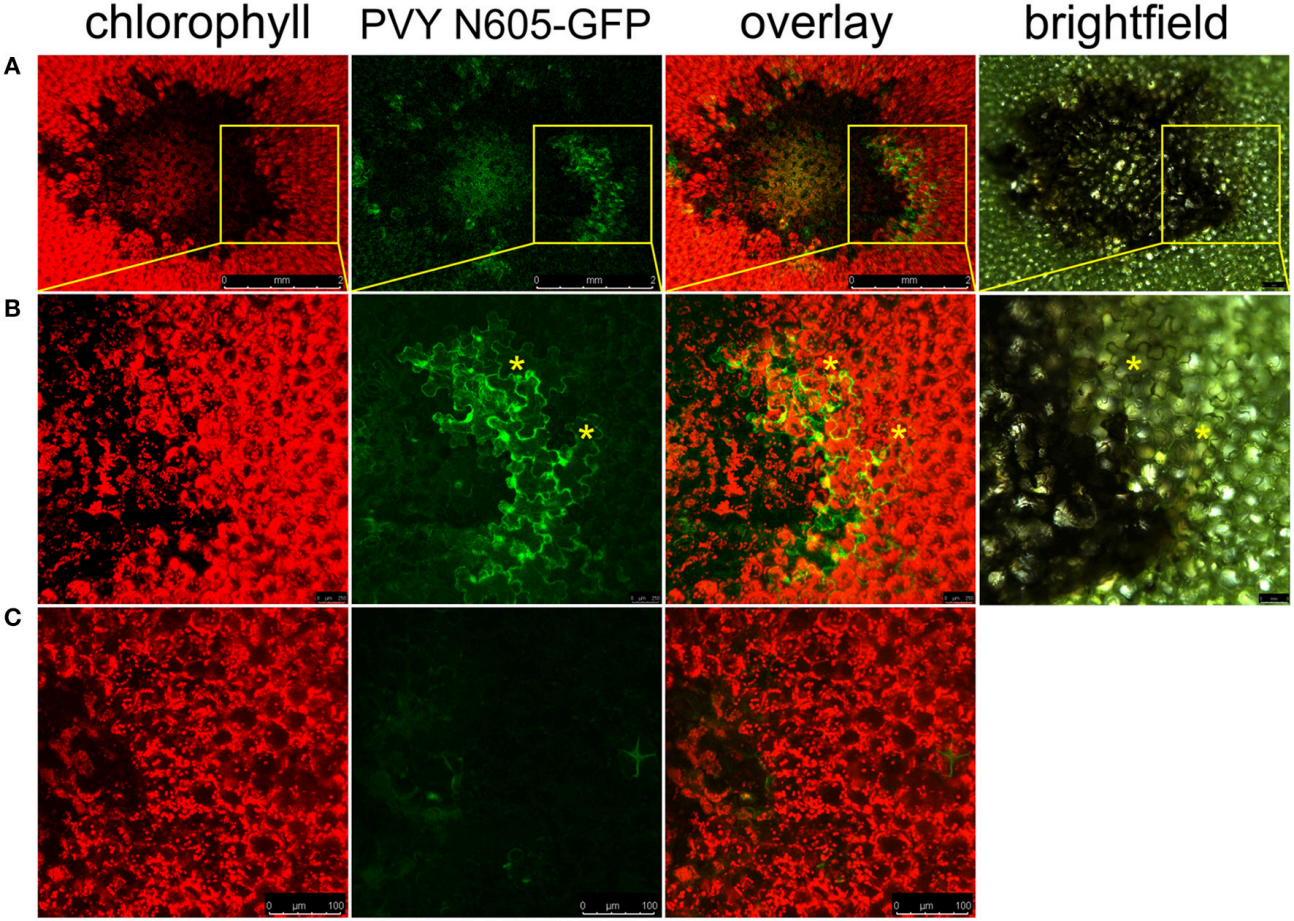
PVY is located outside the cell death zone in potato cv. Rywal. Lesions were scanned by laser scanning confocal microscopy. **(A)** PVY N605-GFP accumulation as observed at 7 dpi. From left to right: chlorophyll fluorescence (red), PVY N605-GFP accumulation (green), overlay of chlorophyll fluorescence and PVY N605-GFP accumulation (scale bars 2 mm), brightfield (scale bar 500 μm). **(B)** High-magnification of the boxed areas in **(A)** (scale bars 250 μm for confocal microscopy and 500 μm for brightfield). **(C)** Shows a lesion after inoculation with PVY without GFP tag as a control (scale bars 100 μm). Asterisks indicate two representative infected cells located outside the cell death zone. Chlorophyll and GFP fluorescence are presented as maximum projections of z-stacks.

To study the link between resistance and cell death in *Ny-1* resistance-gene-mediated response, we infected potato plants of cv. Rywal with a GFP-tagged PVY (PVY N605-GFP). In this infective clone, GFP was inserted between NIb and CP coding regions and was flanked by protease recognition sites, allowing excision from the polyprotein and release into the cytoplasm after translation (Rupar et al., 2015). In our pathosystem, the virus was arrested in the inoculated leaves and could not spread systemically. This was determined by the absence of the symptoms and viral RNA in systemic leaves (Supplementary Table 3). In order to investigate the possible spread of the virus around the lesion, we scanned the area around the cell death zone in PVY N605-GFP inoculated leaf. We observed individual cells or small clusters of cells expressing GFP at the margins of some of the analyzed lesions (Figure 1).

**Figure 2:**
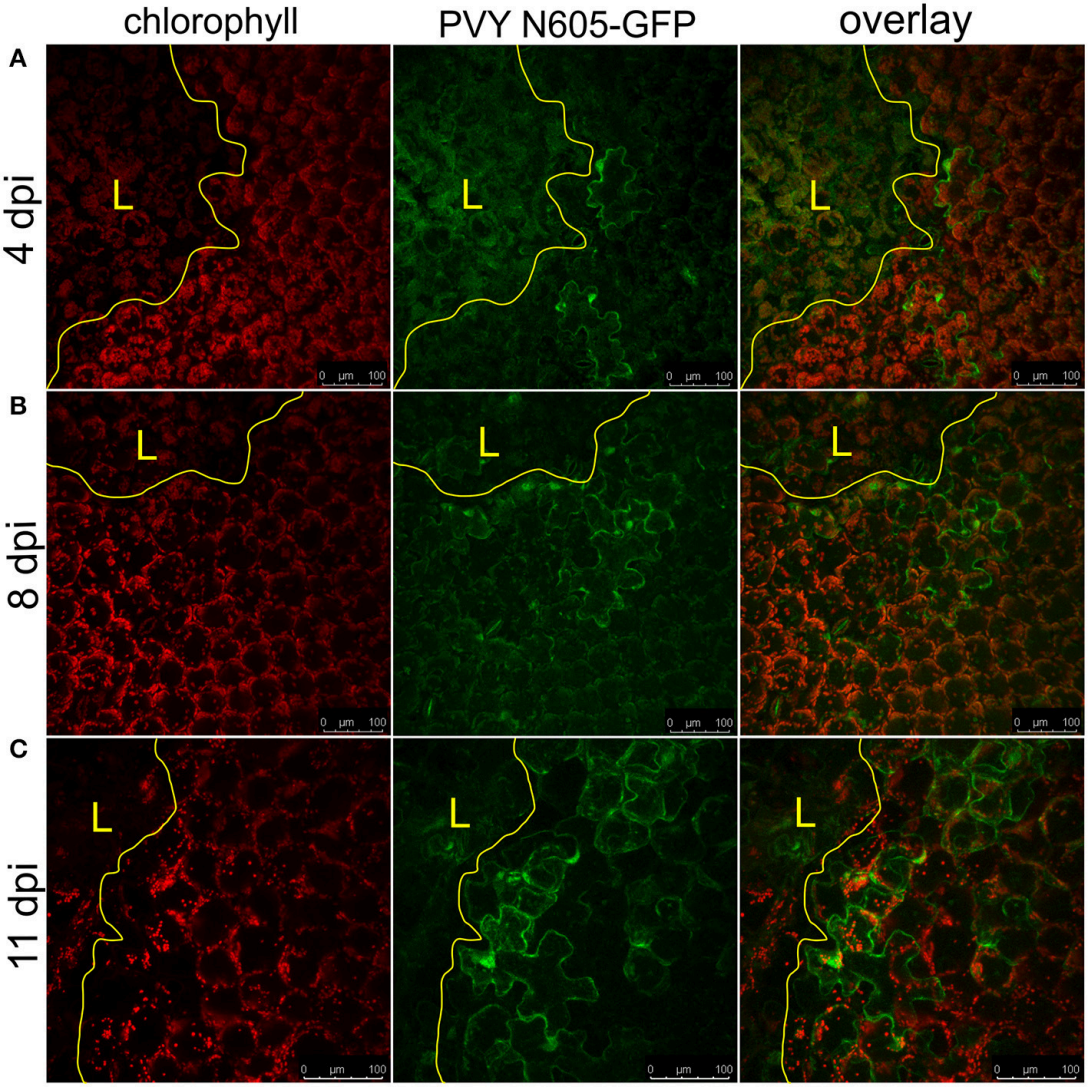
PVY was detected outside the cell death zone in cv. Rywal at different time points following infection. Lesions at 4 dpi **(A)**, 8 dpi **(B)**, and 11 dpi **(C)** are shown. From left to right: chlorophyll fluorescence (red), PVY N605-GFP accumulation (green), overlay of chlorophyll fluorescence and PVY N605-GFP accumulation. PVY N605-GFP accumulation was detected outside some of the lesions at all analyzed time points after inoculation (see Supplementary Table 1). The lesion is marked by the yellow L and the edge of the cell death zone is marked by the yellow line. Scale bars are 100 μm.

We further investigated the occurrence of PVY N605-GFP infected cells outside the cell death zone in cv. Rywal at different time points after inoculation. PVY N605-GFP accumulation was detected outside some of the lesions at all analyzed time points (Supplementary Table 1). However, the number of cells in which the PVY N605-GFP accumulation was observed was similar at all time points from 3 to 11 dpi (Figure 2, Supplementary Table 1).

### Virus spread is continuous in SA depleted transgenic counterparts

**Figure 3:**
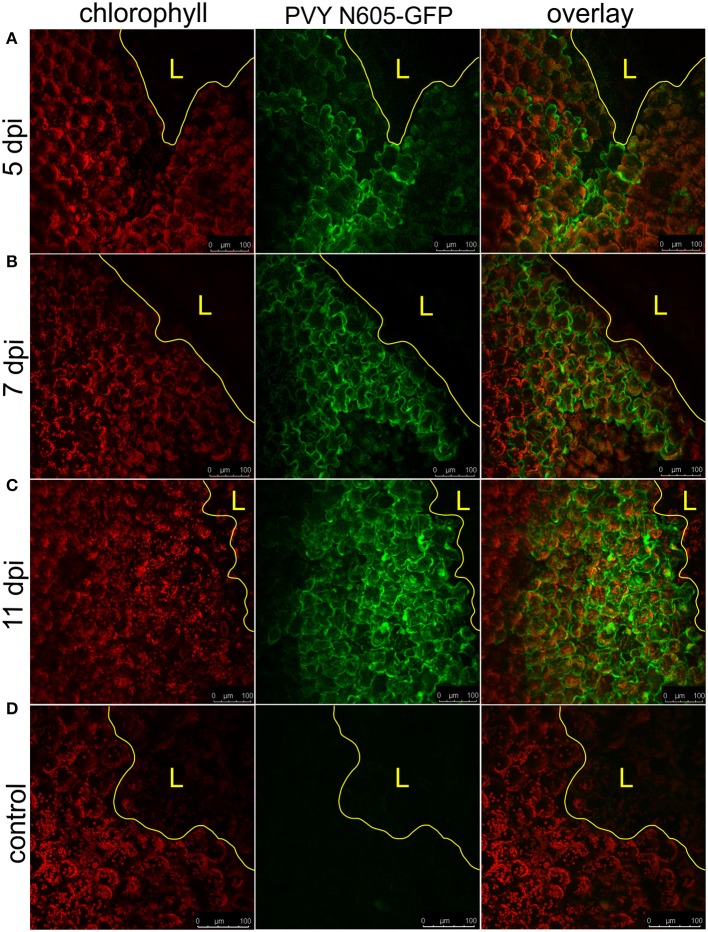
PVY spread around the cell death zone in NahG-Rywal transgenic plants. Lesions at 5 dpi **(A)**, 7 dpi **(B)**, and 11 dpi **(C)** are shown. **(D)** Shows a lesion inoculated with PVY without GFP tag. From left to right: chlorophyll fluorescence (red), PVY N605-GFP accumulation (green), overlay of chlorophyll fluorescence and PVY N605-GFP accumulation. The number of the cells around the cell death zone in which PVY N605-GFP accumulation was detected was higher at later time points after inoculation. The lesion is marked by the yellow L and the edge of the cell death zone is marked by the yellow line. Scale bars are 100 μm.

NahG-Rywal transgenic plants are unable to restrict the virus close to the site of inoculation, which leads to the systemic spread of the virus (Baebler et al., 2014). Therefore, we investigated them in a similar setup to study the dynamics of virus spread around the lesions in the non-resistant host. We followed the PVY N605-GFP accumulation in the cells around the cell death zone from 3 to 12 dpi by confocal microscopy. As expected, we observed the PVY N605-GFP accumulation outside some of the lesions at all analyzed time points from 4 dpi on (Figure 3, Supplementary Table 1). We were unable to detect PVY N605-GFP accumulation at the time of first lesion occurrence (3 dpi) or before lesion formation. The number of the epidermal cells around the cell death zone in which the PVY N605-GFP accumulation was observed, was increasing from 4 to 11 dpi (Figure 3). Beside the epidermal cells, also the number of the GFP-containing mesophyll cells was increasing from 4 to 11 dpi, confirming the concentric spread of the virus, as already observed by Rupar et al. (2015).

### Virus is outside the cell death zone in all lesion developmental stages in HR-conferred resistance

**Figure 4:**
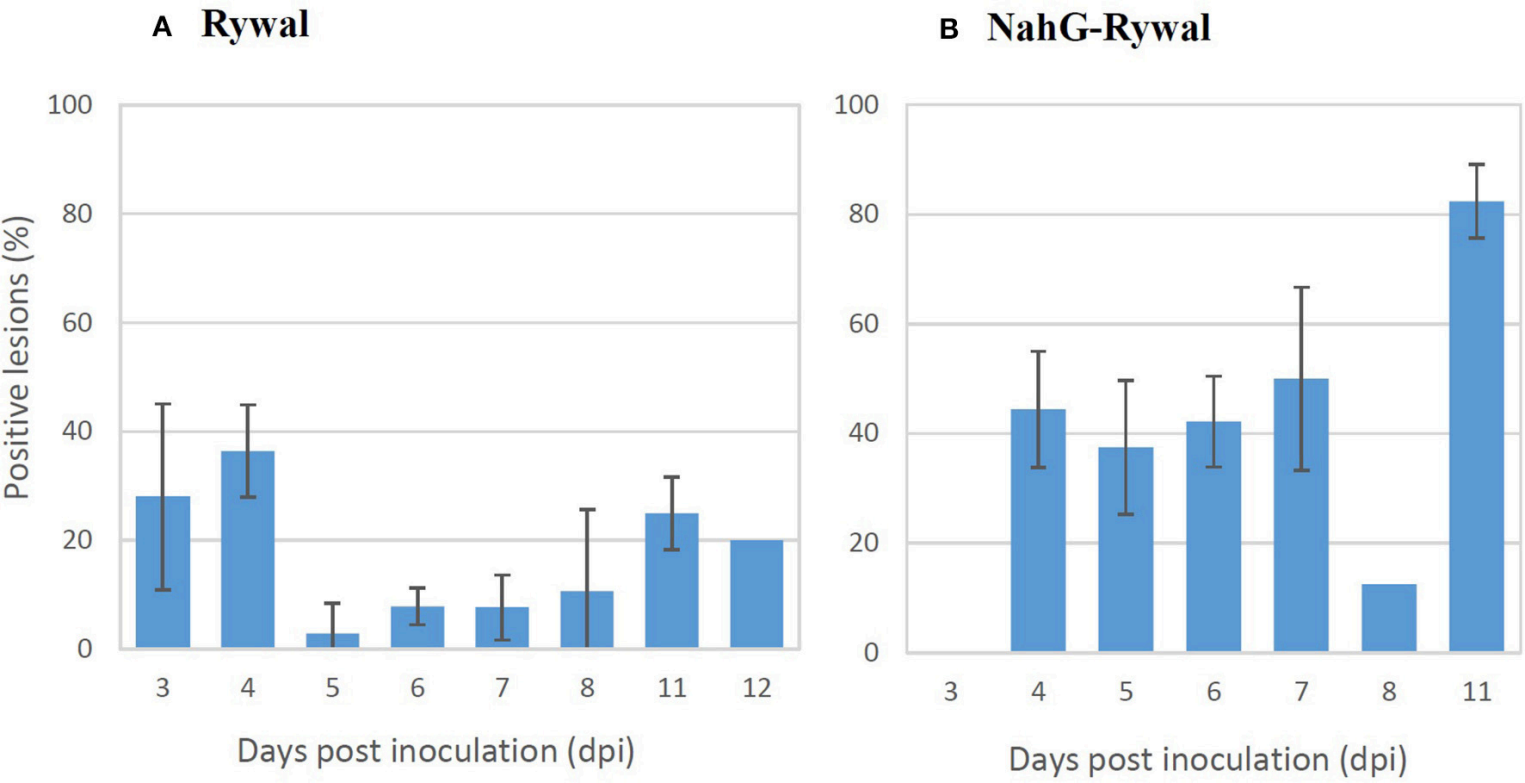
Percentage of lesions with detected PVY outside the cell death zone. Rywal and NahG-Rywal plants were inoculated with PVY N605-GFP to follow PVY N605-GFP accumulation in the cells around the cell death zone on different days post inoculation (dpi). The percentage of positive lesions was calculated as the number of lesions with PVY N605-GFP accumulation detected in at least one cell outside the cell death zone, divided by the number of all analyzed lesions. Error bars represent the standard error of repeated measurements (*n* = 2–7). Data of individual experiments are available in the Supplementary Table 1.

The percentage of lesions with detected PVY N605-GFP accumulation outside the cell death zone (positive lesions) was determined for both genotypes at different time points following inoculation (Figure 4). PVY N605-GFP accumulation was detected outside the cell death zone for some of the lesions at all time points after inoculation. In cv. Rywal, the percentage of positive lesions was higher at earlier time points (3 and 4 dpi). In NahG-Rywal, the percentage of positive lesions increased over time, reaching more than 80% at 11 dpi.

**Figure 5:**
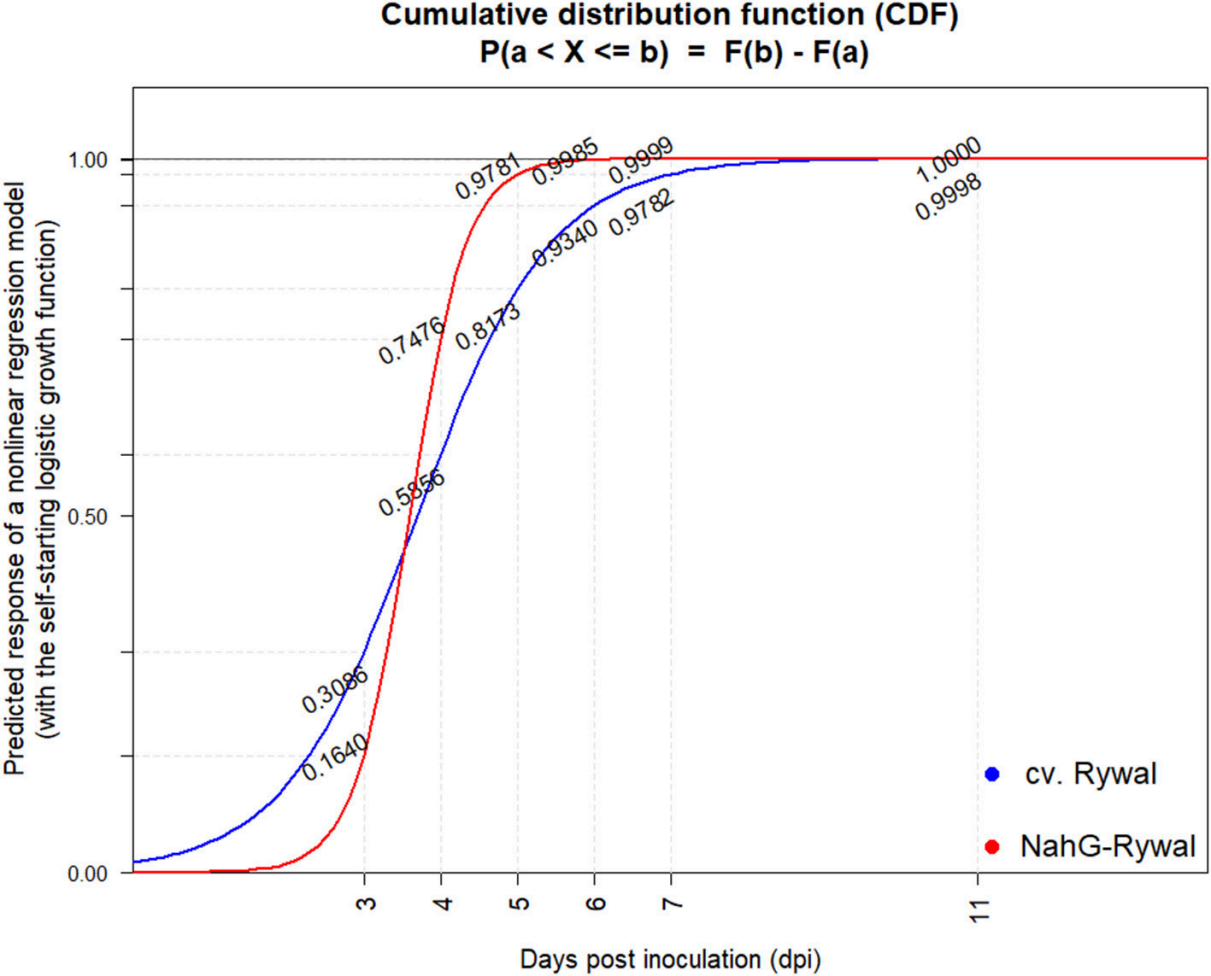
Cumulative probability distribution functions of lesion appearance after PVY inoculation. Lesions were counted at 3, 4, 5, 6, 7, 9, and 11 dpi on all the three inoculated leaves (Figure S3) after PVY N605-GFP inoculation in potato cv. Rywal and NahG-Rywal. To determine the probability that the observed lesion is formed until the particular dpi, cumulative probability distribution function was determined for Rywal (blue) and NahG-Rywal (red). X-axis: dpi, y-axis: probability that the randomly observed lesion is formed until the particular dpi.

In order to exclude the possibility that the lesions analyzed at later time points after inoculation are newly formed lesions, while the virus is successfully enclosed in older lesions, we studied dynamics of lesions development. Results showed that the probability that the lesion is formed after 7 dpi is <2% for both genotypes (Figure 5, Supplementary Table 2). On the other hand, experimental results showed that the virus was present outside the cell death zone in all lesion developmental stages and the percentage of positive lesions at 8, 11, and 12 dpi was at least 10% (Figure 4). As the percentage of positive lesions observed under confocal microscope after 7 dpi was at least 5-times higher than the probability that the lesion is formed after 7 dpi, we can conclude that the virus is present outside the cell death zone in all developmental stages of lesions.

### Cell death zone is continuously expanding in HR-conferred resistance

In order to determine the dynamics of lesion expansion, we followed the lesions over the longer time period by digital microscope until the lesions expanded into large necrotic lesions covering the majority of the leaf (NahG-Rywal) or the senescence occurred (cv. Rywal). In NahG-Rywal, we observed unlimited expansion of all analyzed lesions (Supplementary Video 1, Figure S1). Although the lesion expansion was hardly visible by eye in cv. Rywal at later dpi, using digital microscopy we were able to detect that the majority of analyzed lesions continuously expanded even at later time points (Supplementary Video 2, marked with black arrows in Figure S2). However, compared to NahG-Rywal, the expansion was much slower. On the other hand, some of the analyzed lesions became fully developed already at early time points after inoculation in cv. Rywal (marked with red arrows in Figure S2).

### Virus-infected cells at the periphery of the necrotic lesions are able to reinitiate infection

**Figure 6:**
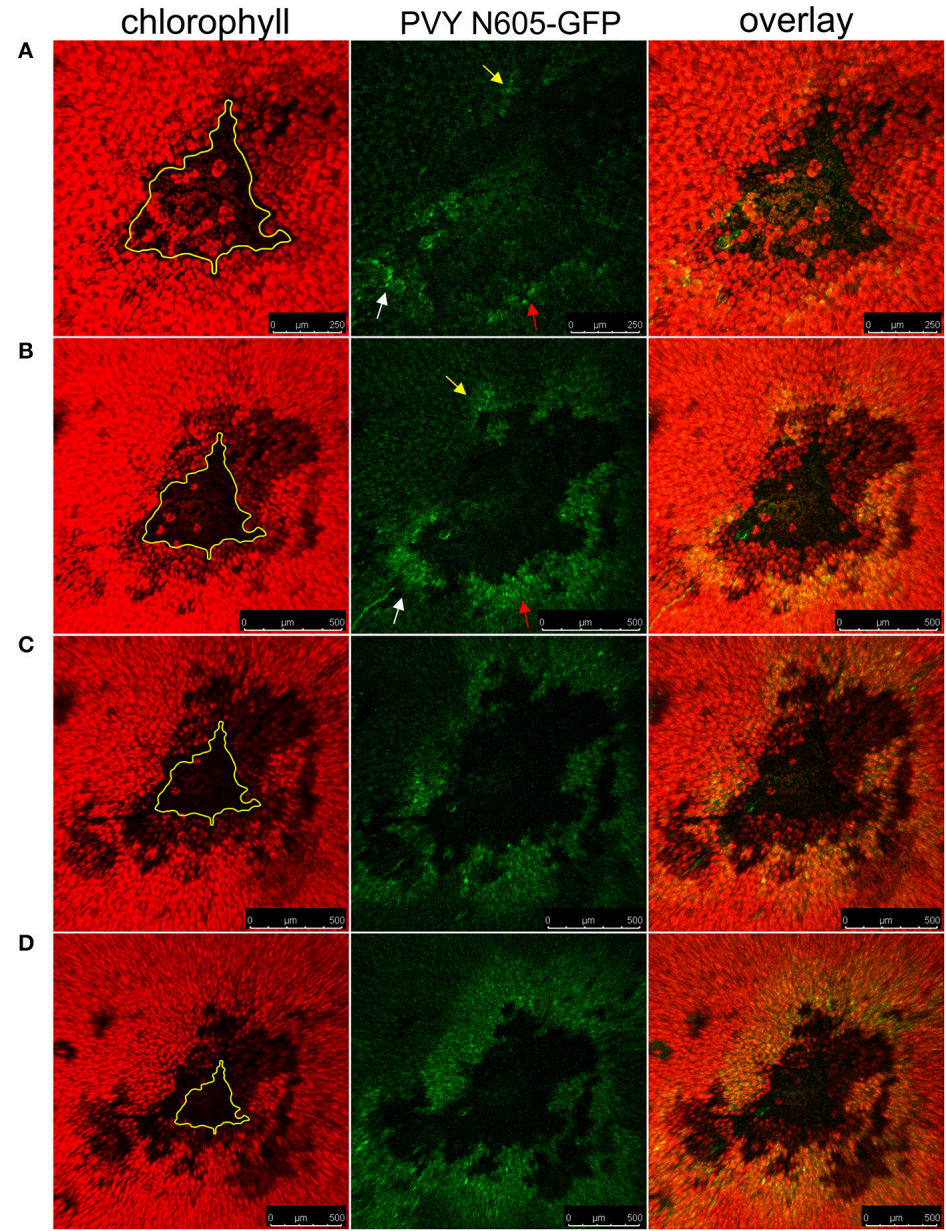
The spread of the PVY following temperature shift in cv. Rywal. Plants were kept at 22°C to allow lesion formation. At 4 dpi, the inoculated leaf was detached, cut into pieces, placed on 1.5% (w/v) agar plate and kept at 22°C. At 4 dpi **(A)**, 5 dpi **(B)**, 6 dpi **(C)**, and 7 dpi **(D)**, the plate was transferred to 28°C for 2 h for confocal microscopy imaging. The higher temperature was used as a condition under which multiplication and spread of the virus are not inhibited by *Ny-1* gene, while the lower temperature as a means to visualize the lesion expansion due to *Ny-1* gene activity. From left to right: chlorophyll fluorescence (red), PVY N605-GFP accumulation (green), overlay of chlorophyll fluorescence and PVY N605-GFP accumulation. The number of GFP-containing cells around the cell death zone was higher at later time points after inoculation confirming the spread of the PVY N605-GFP. The size of the lesion at 4 dpi is indicated by the yellow line. Arrows indicate the development of secondary infection foci. Scale bars are 250 μm **(A)** or 500 μm **(B–D)**.

The *Ny-1*-gene mediated HR-conferred resistance is temperature dependent, as the higher temperature (28°C) leads to abortion of resistance manifested as lack of lesion formation and systemic spread of the virus (Szajko et al., 2008). To test whether the GFP-containing cells at the periphery of the necrotic lesions contain the PVY N605-GFP, which is able to reinitiate infection, we exploited this feature in our system. The temperature shift facilitated the spread of PVY N605-GFP, initiating from the infected cells at the cell death zone margin, and resulted in the development of secondary infection foci, as the number of GFP-containing cells around the cell death zone was higher at later time points after inoculation (Figure 6). We confirmed that the cells at the periphery of the cell death zone are alive and contain the PVY N605-GFP, which is able to reinitiate infection.

### Virus can be found outside the formed callose ring

**Figure 7:**
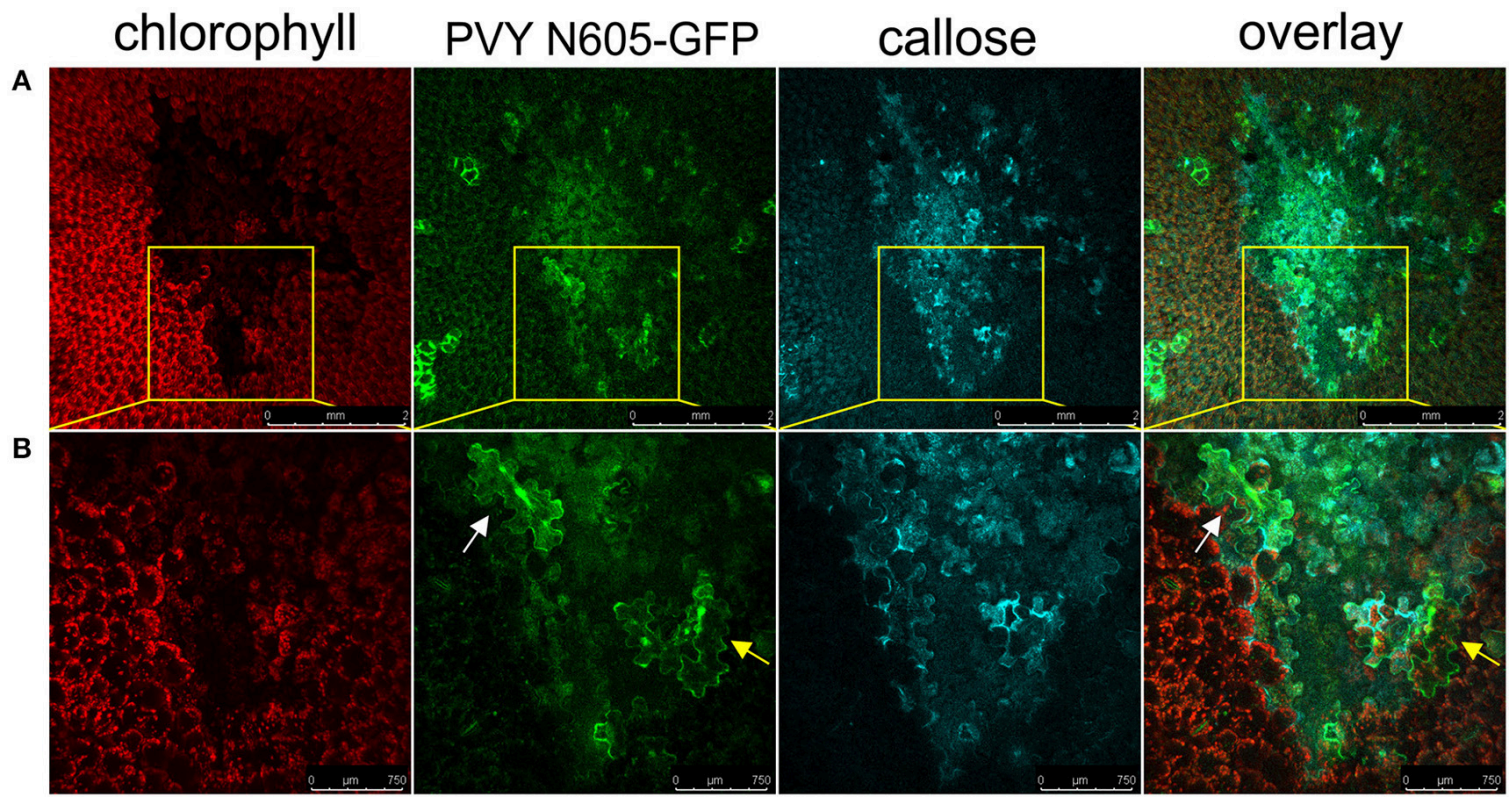
Virus can be found outside the formed callose ring. **(A)** PVY was detected outside the callose deposits at 6 dpi. **(B)** High-magnification of the boxed areas in **(A)** (scale bars are 750 μm). From left to right: chlorophyll fluorescence (red), PVY N605-GFP accumulation (green), callose (blue), overlay of all three channels (scale bars are 2 mm). The infected cell inside the callose ring is shown by the white arrow, while the infected cell outside the callose ring is shown by the yellow arrow.

We next investigated if the callose deposition is crucial for the virus restriction at the periphery of the necrotic lesions in cv. Rywal. Callose deposits were detected at the margin between the cell death zone and the tissue outside the cell death zone in the majority of analyzed lesions forming a continuous ring around the cell death zone (Figure 7). In some of the analyzed lesions, the callose was also accumulated inside the cell death zone and rarely outside the cell death zone. The PVY N605-GFP accumulation was detected inside (white arrow) but in some cells also outside the callose ring (yellow arrow, Figure 7) This indicates that the callose is either not blocking the spread of the virus to cells adjacent of necrotic lesions or that callose deposition at levels below our detection limit is already sufficient for its function.

## Discussion

Traditionally, HR cell death was envisioned as crucial for disease resistance in host-pathogen interaction, which results in restriction of pathogen growth and spread. This notion was first challenged in 1972, when Király et al. suggested, that it is not the necrosis of host tissues which inhibits or prevents the pathogen from further growth, but before necrosis, unknown mechanisms inhibit or even kill the pathogen. The necrosis observed in HR was suggested to be the result of the death or inhibition of the growth of the pathogen, rather than a direct defense mechanism (Király et al., 1972). Later on, however, it became clear that the relative importance of cell death in this form of disease resistance may vary depending on the specific pathosystem (Yu et al., 1998).

In plant-virus pathosystems, the cell death was shown to be uncoupled from resistance in the HR in different host-virus interactions including viruses from several genera, such as genus *Potexvirus* (PlAMV and PVX), *Caulimovirus* (CaMV), *Tombusvirus* [*Tomato bushy stunt virus* (TBSV)], *Cucumovirus* (CMV), *Carmovirus* (TCV), and *Tobamoviruses* [TMV and *Tomato mosaic virus* (ToMV)] (Chu et al., 2000; Chandra-Shekara et al., 2004; Cawly et al., 2005; Ishibashi et al., 2007; Komatsu et al., 2010; Liu et al., 2010; Hafez et al., 2012; Ando et al., 2014). Our study is the first showing similar results also for the interaction of member of another virus family, *Potyviridae*, with its host. However, besides our study, only the studies investigating tobacco–TMV interaction were focused on the precise localization of the virus in relation to cell death zone. Electron microscopy examination of lesions formed in *Nicotiana glutinosa* after infection with TMV confirmed the presence of the virus just outside the edge of the lesion (Milne, 1966; Da Graça and Martin, 1976), as confirmed also by ELISA (Antoniw and White, 1986). Results of two confocal microscopy studies confirmed the presence of isolated cells or small clusters of cells infected with TMV-GFP (Wright et al., 2000; Murphy and Carr, 2002) or TMV-GUS (Arce-Johnson et al., 1995) at the edge of necrotic lesions. Our results are in agreement with the results studying tobacco-TMV interaction as we have visualized similar pattern of virus escape. As in the case of TMV-tobacco pathosystem (Wright et al., 2000), we also show that virus outside cell death zone is alive and able to reinitiate infection from the cells surrounding the lesion (Figure 6). In previous studies, the temperature shift assay was also exploited to determine the time needed for complete elimination of the TMV in HR interaction with the host. In resistant tobacco plants at least 12 days at 22°C were needed to avoid any virus spread after temperature shift (reviewed in Weststeijn, 1981). These results suggest the presence of the viable virus outside the lesion also in late time points after inoculation, which is in accordance with our results showing that the virus is located outside the lesion at all lesion developmental stages (Figure 4, Supplementary Table 1).

Moreover, we elucidated some additional characteristics of HR-conferred resistance using our pathosystem. To determine whether the cells that harbor viable viral elements are in time also engulfed by cell death zone, we followed the dynamics of lesions expansion over the long time period, until the leaf was completely senescent. We show that in this pathosystem the lesions are first visible at 3 dpi and while some lesions maintain their size after 5 dpi, the expansion of majority of the lesions could be observed even after 10 dpi (Supplementary Video 2, Figure S2). In N-gene resistance-gene-mediated response against TMV infection Wright and coworkers were visually studying potential expansion of lesion in later stages, but were not able to detect it and thus concluded that an alternative mechanism exist that selectively eliminates the remaining infected cells (Wright et al., 2000). In the *Ny-1* resistance-gene-mediated responses the infected cells observed outside the cell death zone can be eventually subjected to cell death. In the SA-depleted plants, both the virus and the cell death zone are spreading faster than in cv. Rywal (Figure 3). Therefore, HR-conferred resistance should be regarded as a process where the dynamics of events is crucial for effectiveness.

One of the common mechanisms that control viral movement by preventing cell-to-cell spread of the virus is plasmodesmata closure, which is often regulated by callose deposition (Lee and Lu, 2011). For example, in plants infected with soybean mosaic virus (SMV), callose deposition was extensive within necrotic viral lesions, but could also be detected outside the lesion, suggesting that callose deposits at plasmodesmata could be an early defense strategy limiting the spread of the virus (Li et al., 2012). Callose deposition has also been reported at plasmodesmata in plants infected with many other viruses, including TMV and PVY (reviewed in Zavaliev et al., 2011). However, these studies did not include precise localization of the infected cells in relation to the callose deposits. Our results show the presence of extensive callose deposition on the margin of the cell death zone, engulfing the majority of infected cells and the outside tissue, but the virus was present also outside these callose deposits (Figure 7), suggesting that the callose deposition is not sufficient for virus restriction. However, we cannot exclude the possibility that the callose is contributing to the resistance in HR in potato, albeit at concentration below the limit of our detection method. Physical blockage of plasmodesmata was also not detected in resistant tobacco-TMV interaction, where results were obtained by studying symplastic continuity with micro-injected dyes, although the involvement of callose deposits in the plasmodesmata closure was not investigated (Wright et al., 2000).

Our results show that the virus is multiplying in the immediate vicinity of the cell death zone at all time points post inoculation, yet we did not observe any virus spread to the neighboring tissues. Although we do not exclude the possibility that the cell death contributes to limitation of viral spread and/or multiplication, it is not sufficient to completely prevent the PVY from spreading to cells adjacent to necrotic lesions. Therefore, we suggest that the HR cell death is separated from the resistance mechanisms also in PVY-potato pathosystem, indicating that this might be a general feature of HR against viruses. The molecular mechanisms restricting virus spread, however, remain unknown.

## Author contributions

TL and KrG: conceived and designed the experiments. TL, KaG, and MP-N: performed the experiments; TL, MP-N, ŠB, MZ, and KrG: analyzed and discussed the results; TL, ŠB, MP-N, KaG, MZ, AC, and KrG: contributed to the writing and the revision of the manuscript.

### Conflict of interest statement

The authors declare that the research was conducted in the absence of any commercial or financial relationships that could be construed as a potential conflict of interest.

## Supplementary material

Figure S1 Dynamics of lesion expansion in NahG-Rywal after PVY N605-GFP inoculation. Lesions expansion was followed by DinoLite digital microscope in 5 independent experiments. We observed unlimited expansion of all analyzed lesions.

Figure S2 Dynamics of lesion expansion in cv. Rywal after PVY N605-GF Pinoculation. Lesions expansion was followed by DinoLite digital microscope in 13 independent experiments. Black arrows indicate some of the lesions which continuously expanded even in later dpi, while red arrows indicate some of the lesions which became fully developed already in early time points after inoculation.

Figure S3 Number of lesions developed on potato leaves after inoculation with PVY N605-GFP. Lesions were counted in 16 cv. Rywal and 11 NahG-Rywal plants on all three inoculated leaves from 3 to 7 dpi and on 11 dpi to study the dynamics of lesions formation. **(A)** Numbers of lesions observed on each plant leaf at each time point is shown. X: detached leaf for confocal microscopy, F: the leaf fell off the plant, /: lesions not counted. Some of the leaves (marked with green in the tables) from this experiment were detached from the plants and used to follow virus spread around the lesions (see exp 2 in Supplementary Table 1). **(B)** Numbers of lesions for each plant leaf from 3 to 11 dpi for selected plants for both genotypes. **(C)** Standardized imputed cumulative sums of lesions on each leaf from 3 to 11 dpi for selected plants (see Methods for data filtering).

Supplementary Table 1 **(A)** Number of lesions with the PVY N605-GFP accumulation detected outside the cell death zone after inoculation. Results from seven independent experiments (Exp 1–7) are presented for cv. Rywal and NahG-Rywal. At each time point (3–12 dpi) one or two plants **(A,B)** were analyzed (one leaf per plant). Number of positive lesions (number of lesions with the PVY N605-GFP accumulation detected outside the cell death zone) on the particular day postinoculation/the number of all analyzed lesions are shown. - denotes that a plant was not analyzed at a particular time point. **(B)** Number of the GFP-containing cells around the positive lesions in cv. Rywal. n.c. denotes that GFP-containing cells around the particular positive lesions were not counted.

Supplementary Table 2 The probability that the observed lesion was formed until the particular day post inoculation. The probability was calculated after PVY N605-GFP inoculation of cv. Rywal **(A)** and NahG-Rywal **(B)** plants by nonlinear regression model with logistic function.

Supplementary Table 3 Relative PVY RNA abundance in the systemic leaves in Rywal and NahG-Rywal plants after PVY inoculation. Relative PVY RNA abundance was determined in upper non-inoculated leaves 4 weeks after inoculation with PVY N605-GFP. Results were obtained from two independent experiments. Relative abundance of PVY RNA was followed using quantitative PCR. RNA was isolated from upper non-inoculated leaves using the RN easy Plant Mini Kit (Qiagen) according to the manufacturer's instructions. DNase-treated (0.5 μl DNase per μg RNA; Qiagen) total RNA (1–2 μg) was reverse transcribed using the High Capacity cDNA Reverse Transcription Kit (Applied Biosystems). Amount of virus RNA was normalized to expression of cytochrome oxidase (Cox) according to Baebler et al. (2011). For the detection of Cox and PVY RNA we used TaqMan chemistry as previously described (Baebler et al., 2011). The standard curve method was used for relative quantification using quant Genius (http://quantgenius.nib.si; Baebler et al., 2017). Under LOD: under limit of detection.

Supplementary Video 1 Dynamics of lesion expansion in NahG-Rywal after PVY N605-GFP infection.

Supplementary Video 2 Dynamics of lesion expansion in Rywal after PVY N605-GFP infection.
