# Supplementary material for: Cell Death Is Not Sufficient for the Restriction of Potato Virus Y Spread in Hypersensitive Response-Conferred Resistance in Potato
Source: Front Plant Sci. 2018 Feb 15;9:168. doi: 10.3389/fpls.2018.00168 (PMC5818463; doi:10.3389/fpls.2018.00168)
Supplement: Figure S1 — Dynamics of lesion expansion in NahG-Rywal after PVY N605-GFP inoculation. Lesions expansion was followed by DinoLite digital microscope in 5 independent experiments. We observed unlimited expansion of all analyzed lesions. [file FigureS1.pdf]

Dynamics of lesion expansion in NahG-Rywal

DinoLite digital microscopy

Supplementary Figure 1: Dynamics of lesion expansion in NahG-Rywal after PVY N605-GFP inoculation. Lesions expansion was followed by DinoLite digital microscope in five independent experiments. We observed unlimited expansion of all analysed lesions.

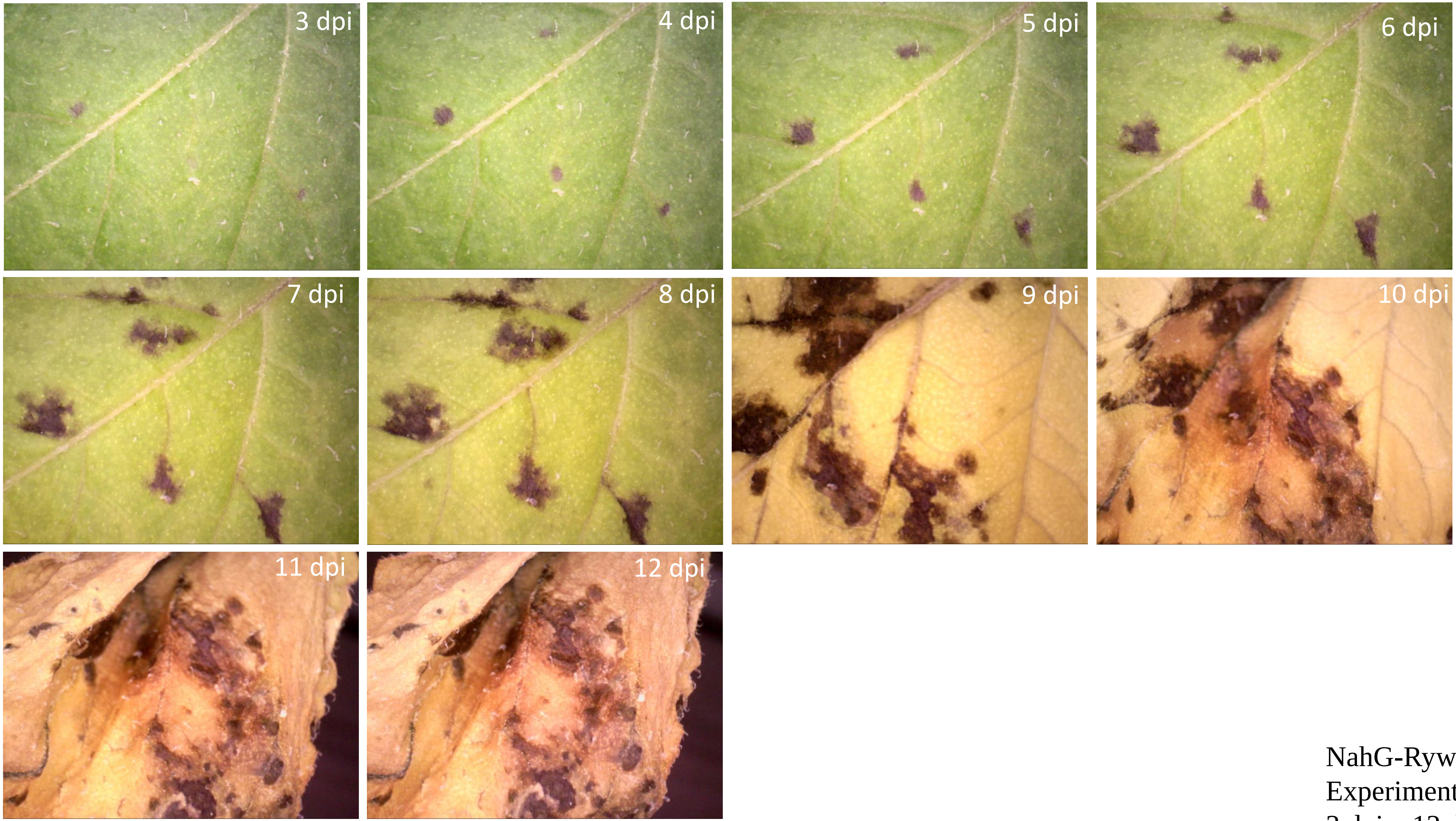

NahG-Rywal
Experiment 1
3 dpi – 12 dpi

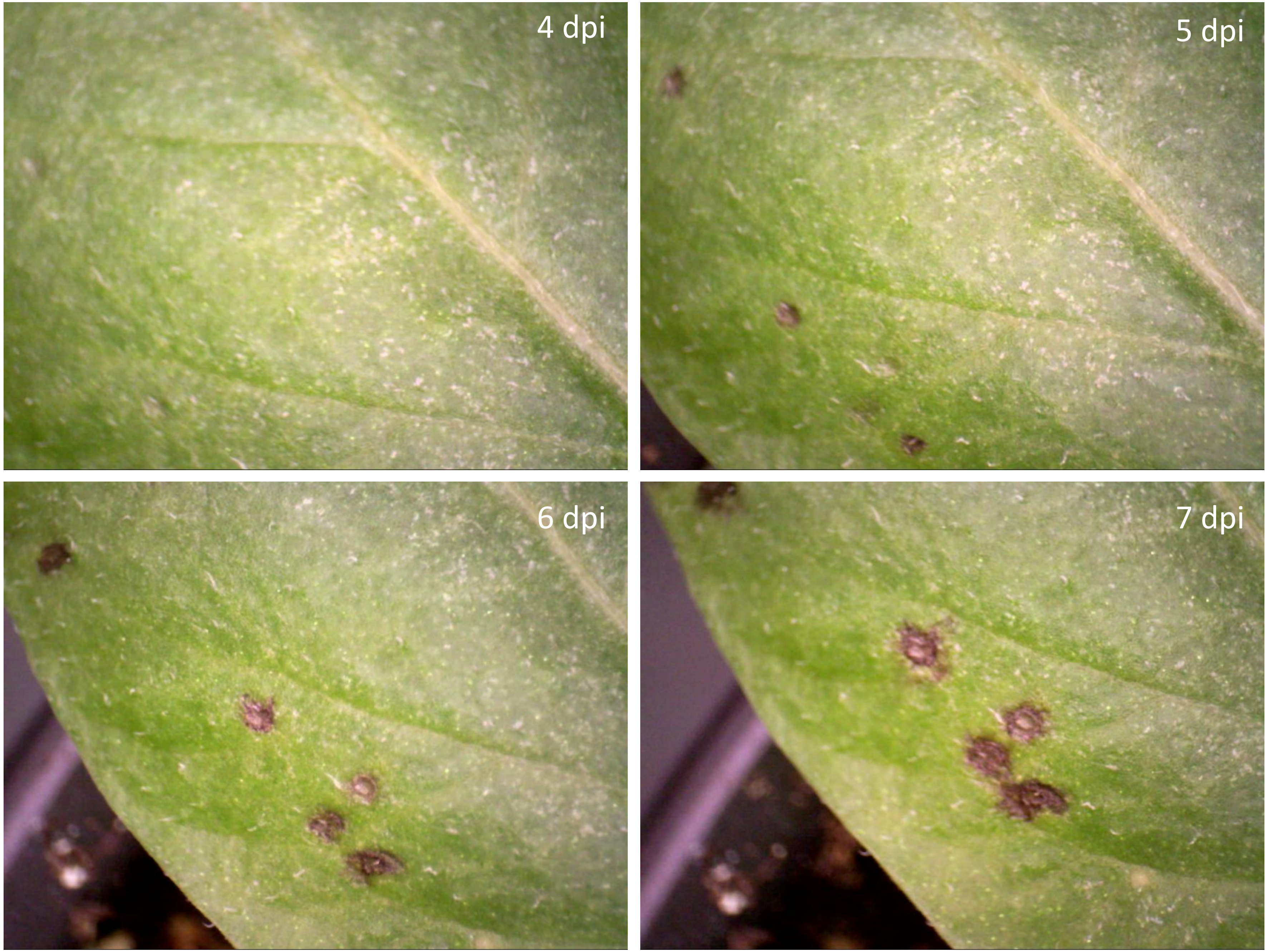

NahG-Rywal
Experiment 2
4 dpi – 7 dpi

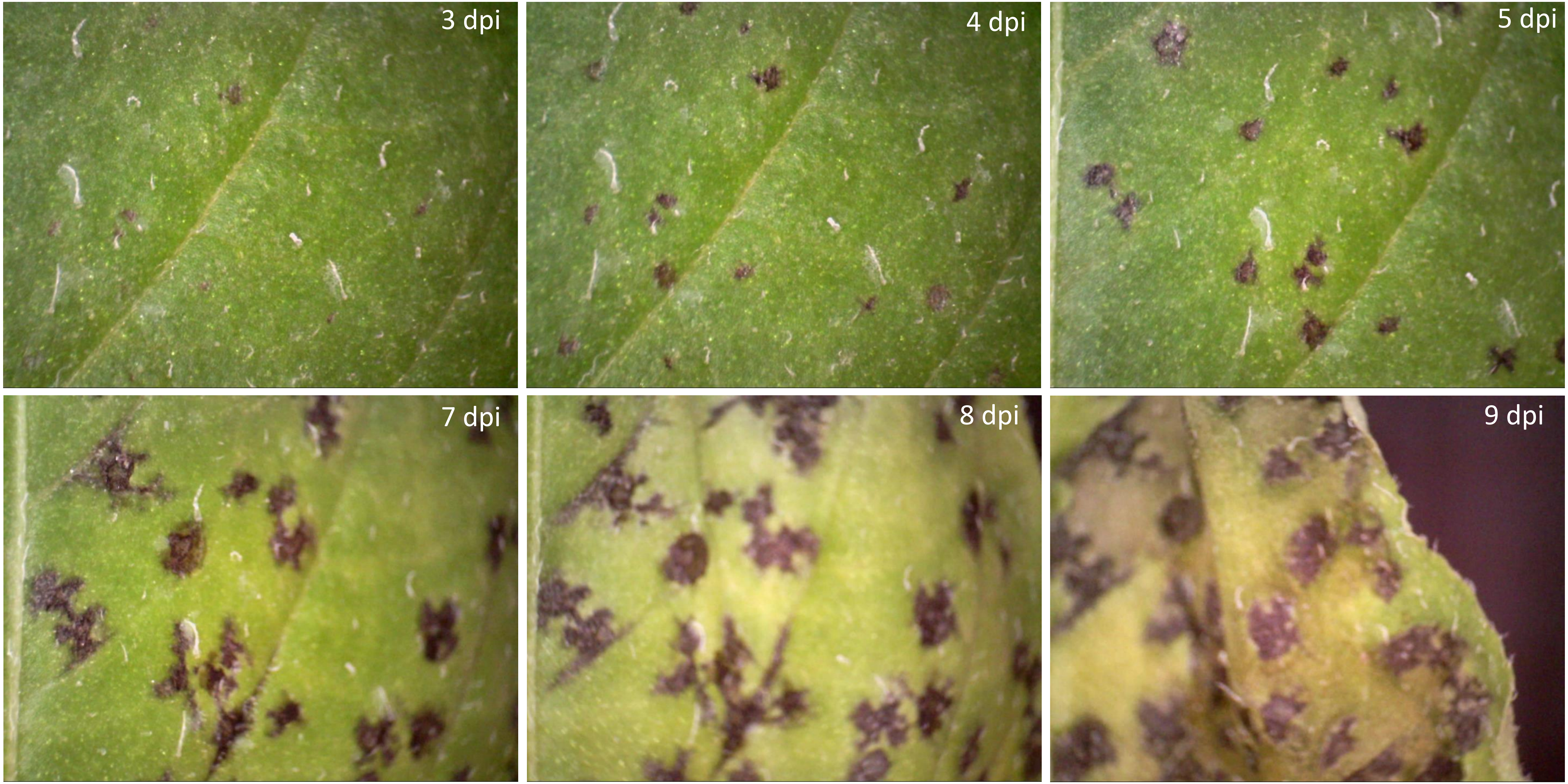

NahG-Rywal
Experiment 3
3 dpi – 9 dpi

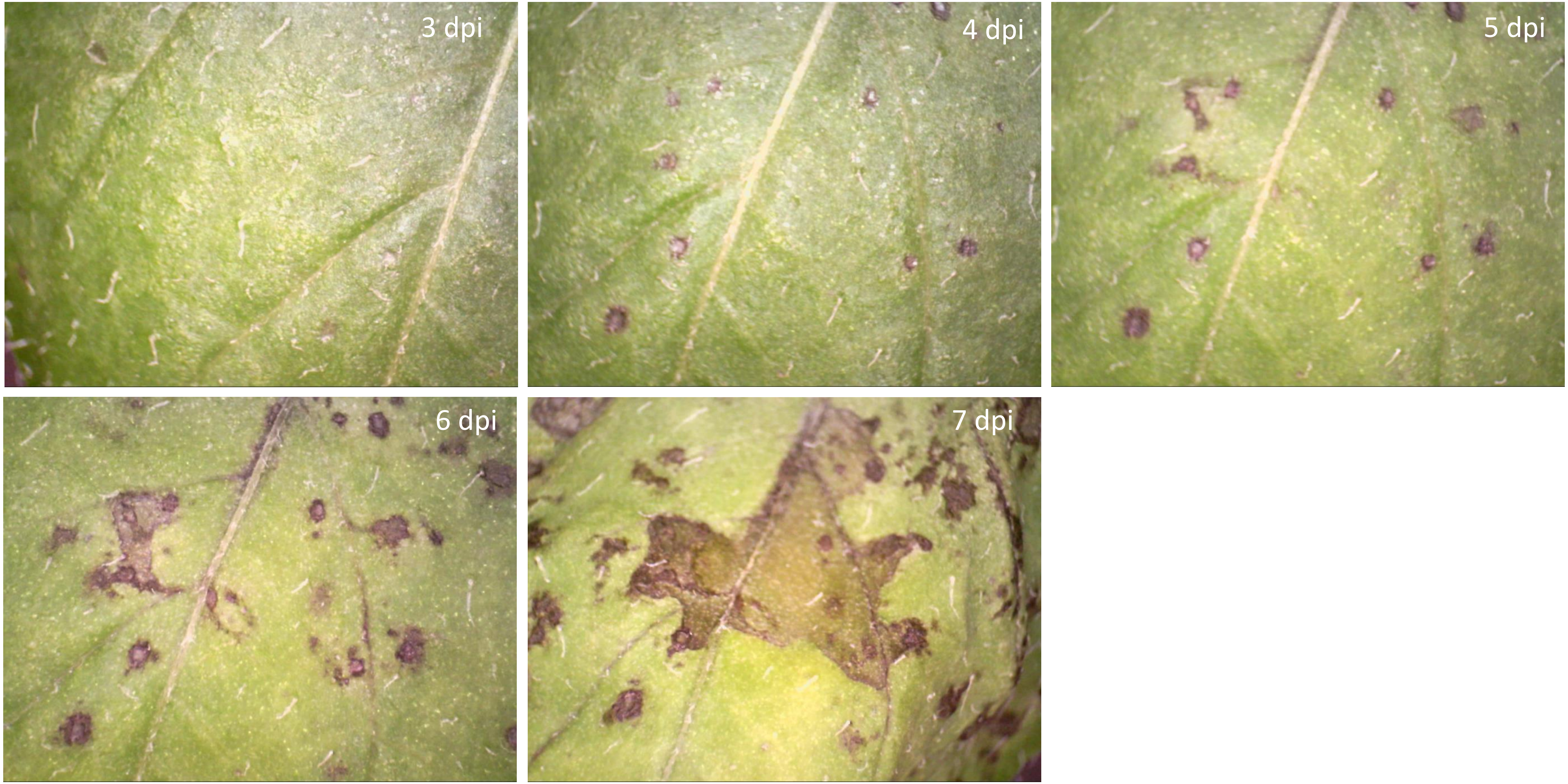

NahG-Rywal
Experiment 4
3 dpi – 7 dpi

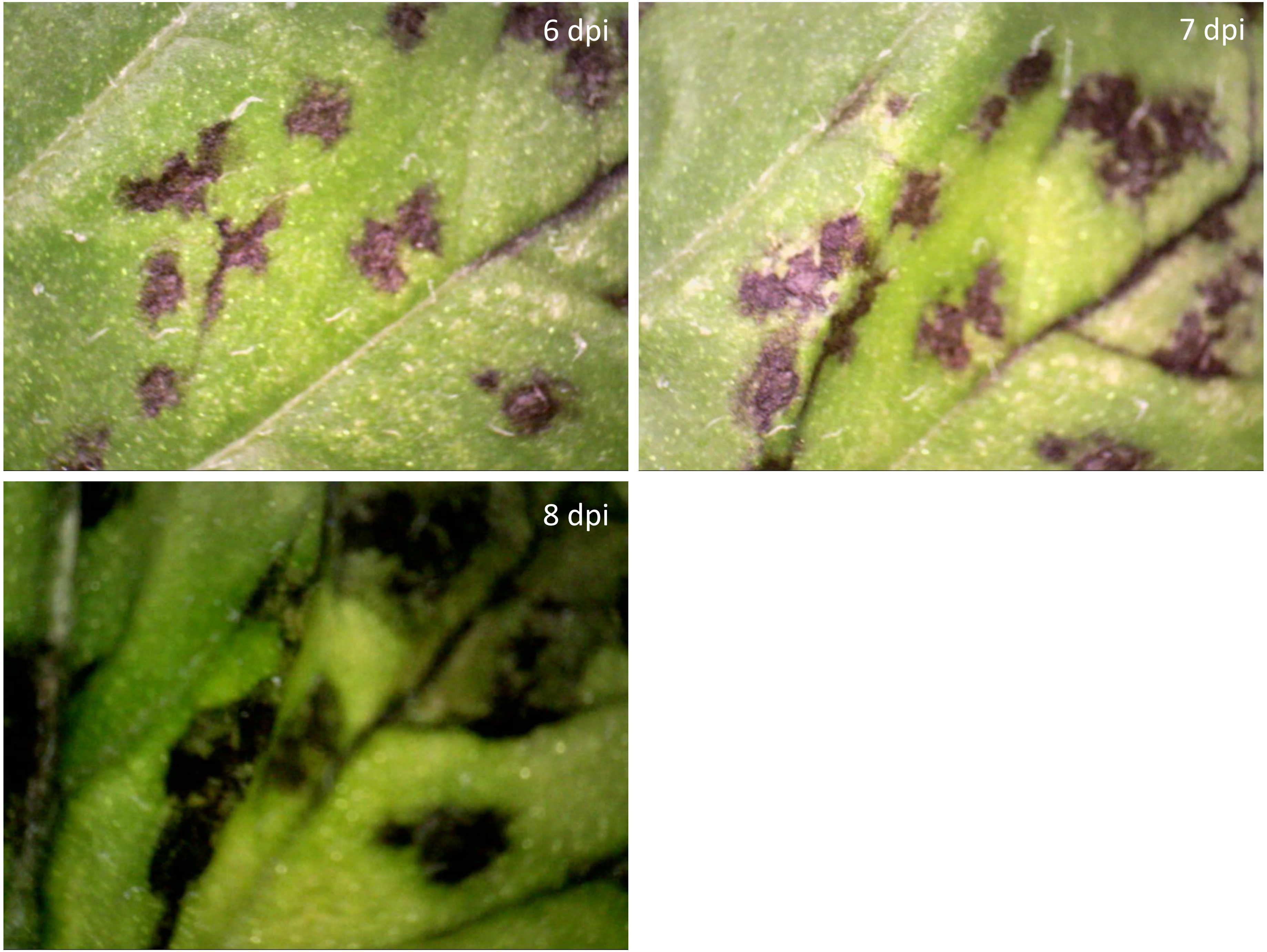

NahG-Rywal
Experiment 5
6 dpi – 8 dpi
