# Supplementary material for: Cell Death Is Not Sufficient for the Restriction of Potato Virus Y Spread in Hypersensitive Response-Conferred Resistance in Potato
Source: Front Plant Sci. 2018 Feb 15;9:168. doi: 10.3389/fpls.2018.00168 (PMC5818463; doi:10.3389/fpls.2018.00168)
Supplement: Figure S2 — Dynamics of lesion expansion in cv. Rywal after PVY N605-GF Pinoculation. Lesions expansion was followed by DinoLite digital microscope in 13 independent experiments. Black arrows indicate some of the lesions which continuously expanded even in later dpi, while red arrows indicate some of the lesions which became fully developed already in early time points after inoculation. [file FigureS2.pdf]

Dynamics of lesion expansion in cv. Rywal

DinoLite digital microscopy

Supplementary Figure 2: Dynamics of lesion expansion in cv. Rywal after PVY N605-GFP inoculation. Lesions expansion was followed by DinoLite digital microscope in 13 independent experiments. Black arrows indicate some of the lesions which continuously expanded even in later dpi, while red arrows indicate some of the lesions which became fully developed already in early time points after inoculation.

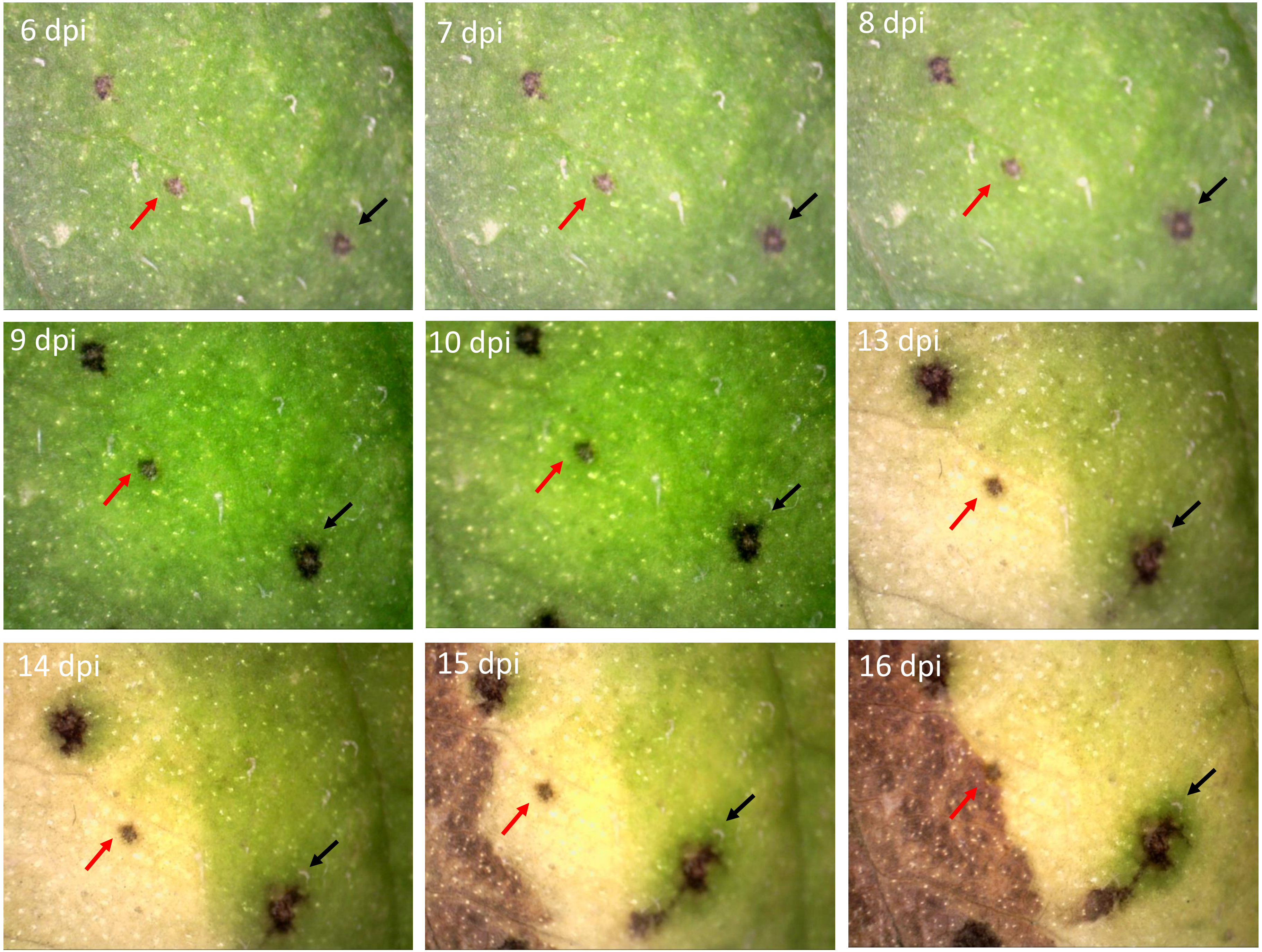

cv. Rywal
Experiment 1
6 dpi – 16 dpi

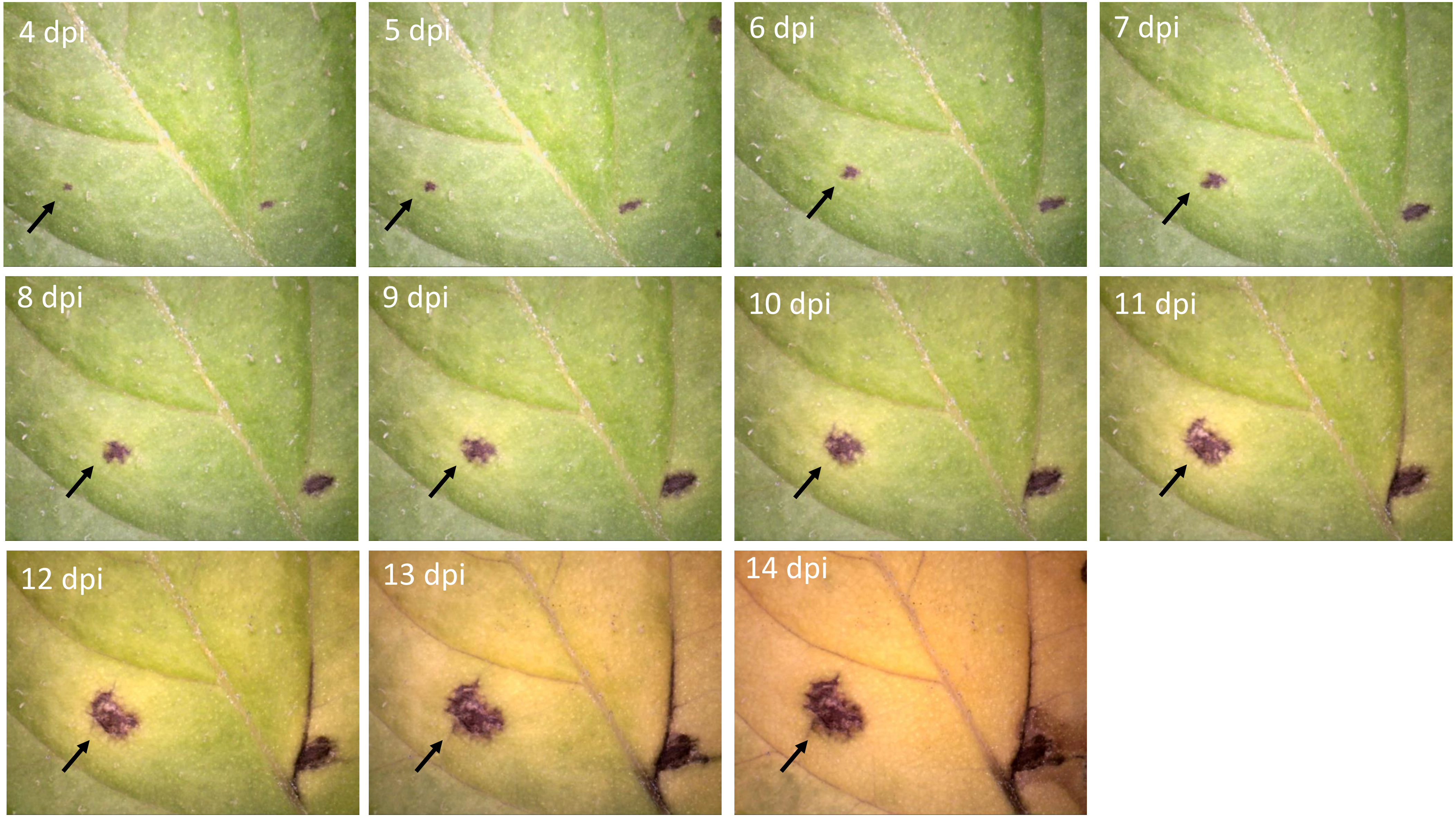

cv. Rywal
Experiment 2
4 dpi – 14 dpi

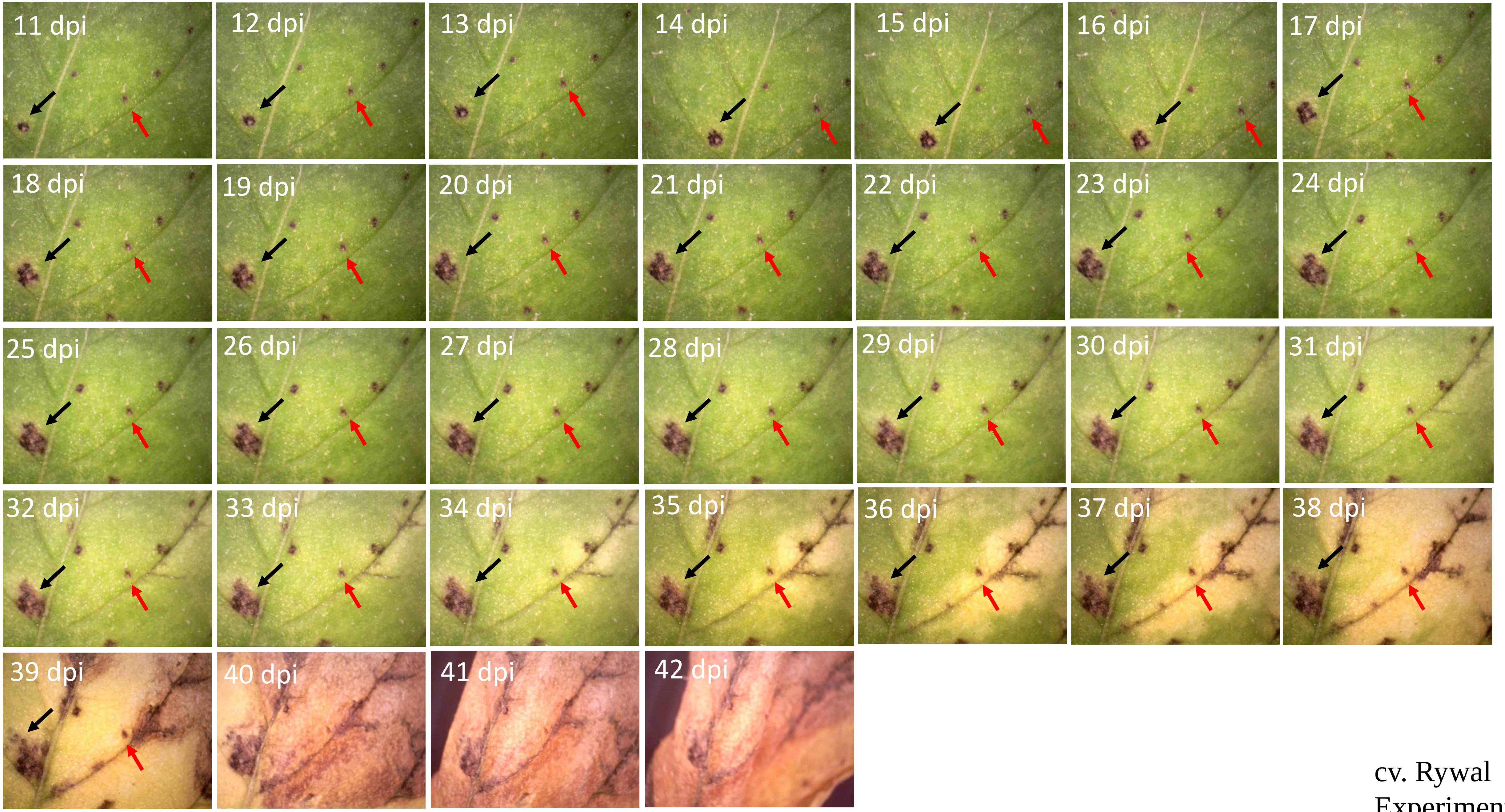

cv. Rywal
Experiment 3
11 dpi – 42 dpi

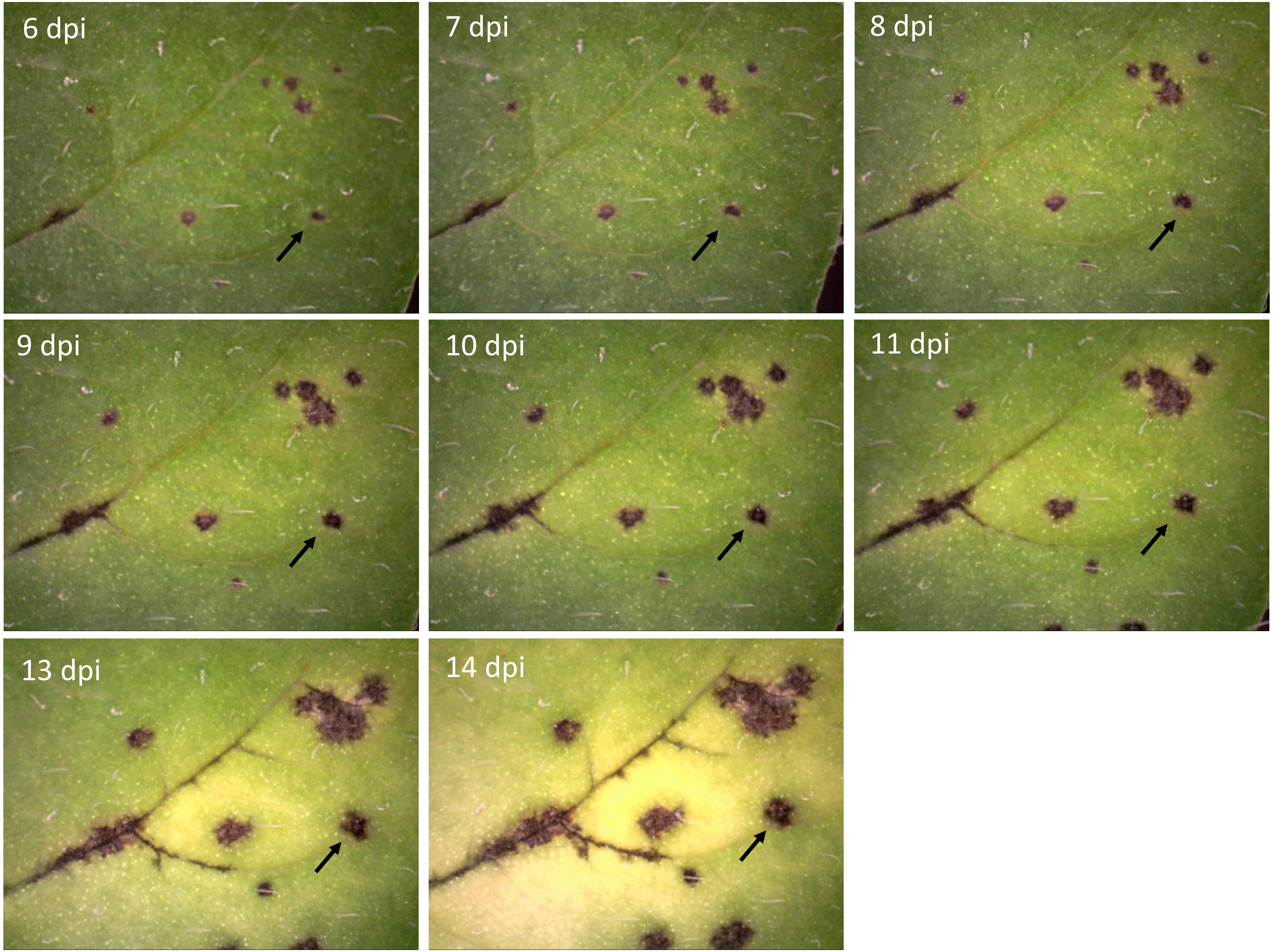

cv. Rywal
Experiment 4
6 dpi – 14 dpi

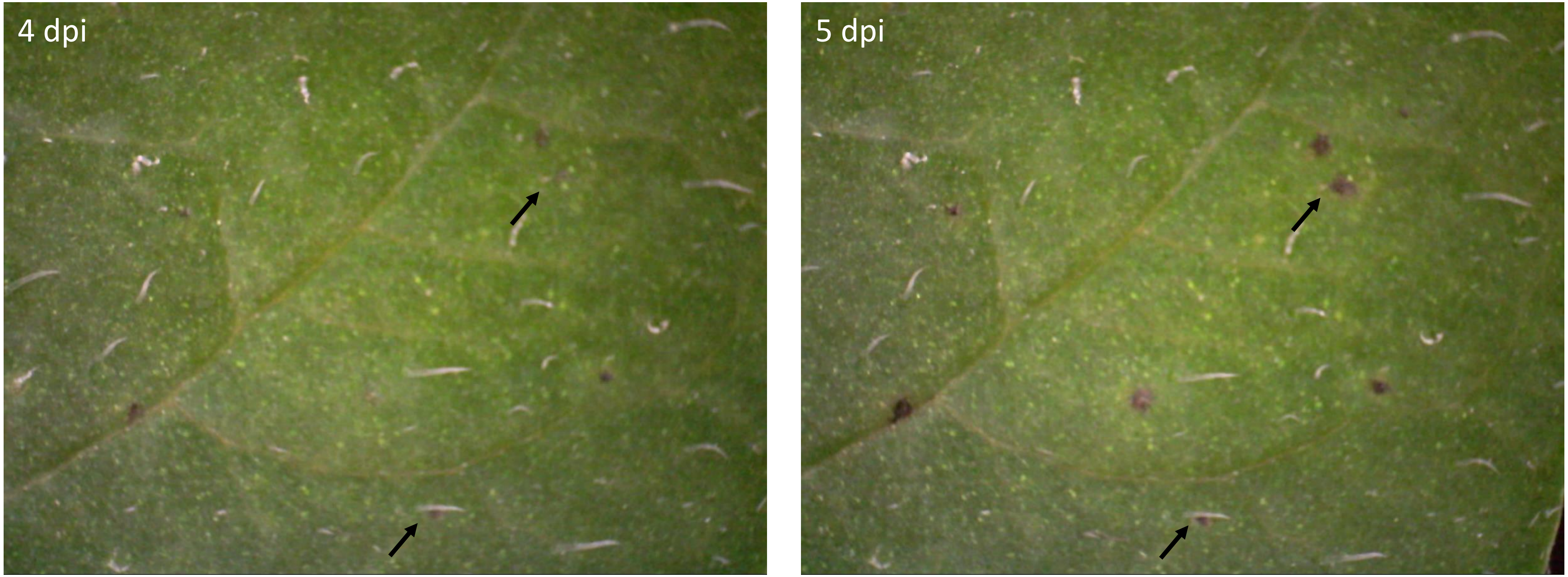

cv. Rywal
Experiment 5
4 dpi – 5 dpi

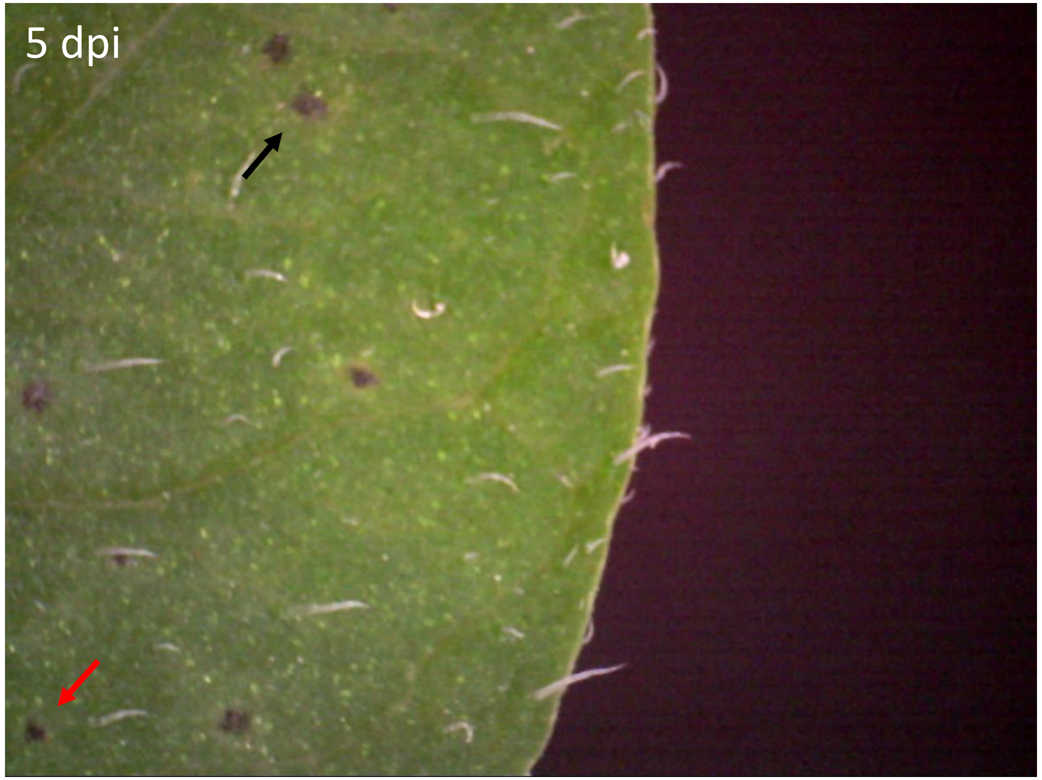

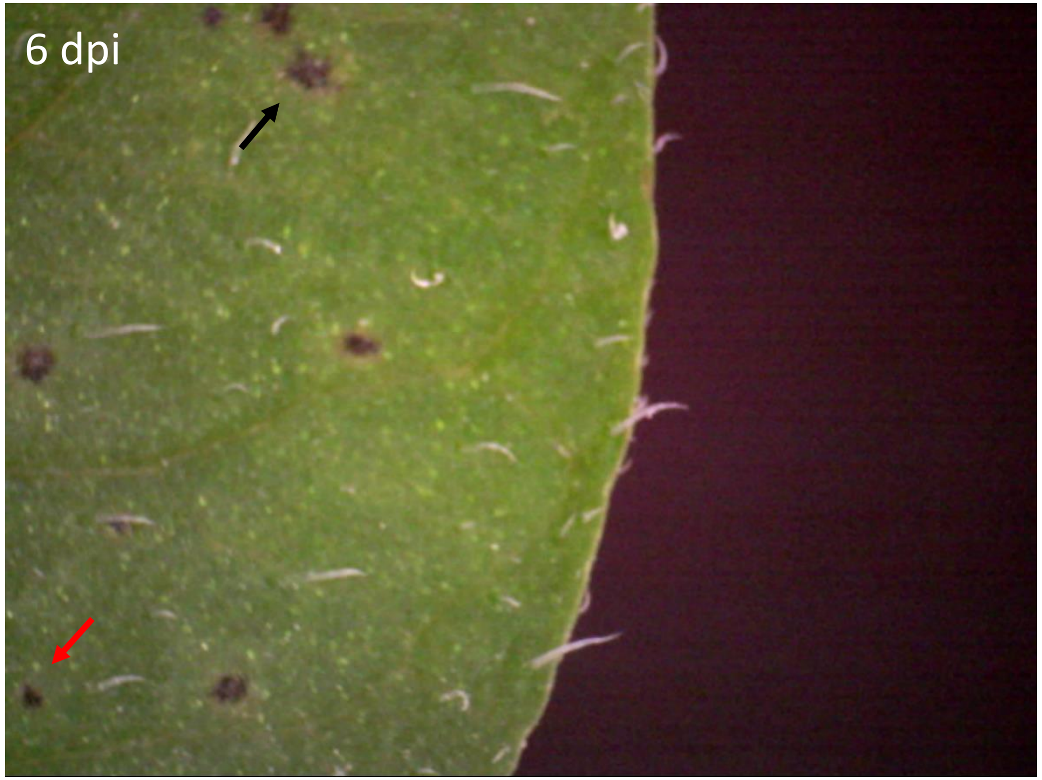

cv. Rywal
Experiment 6
5 dpi – 6 dpi

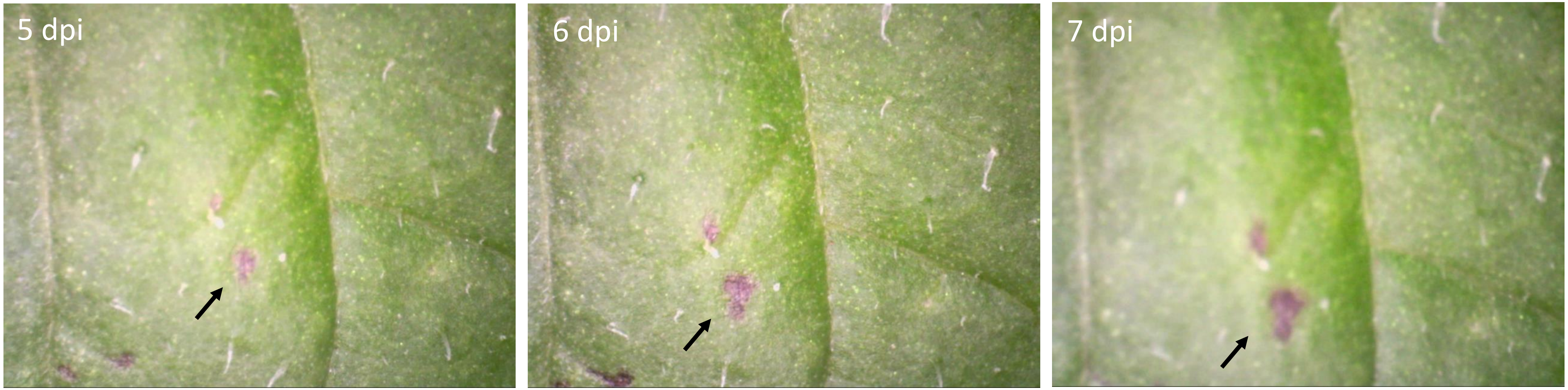

cv. Rywal
Experiment 7
5 dpi – 7 dpi

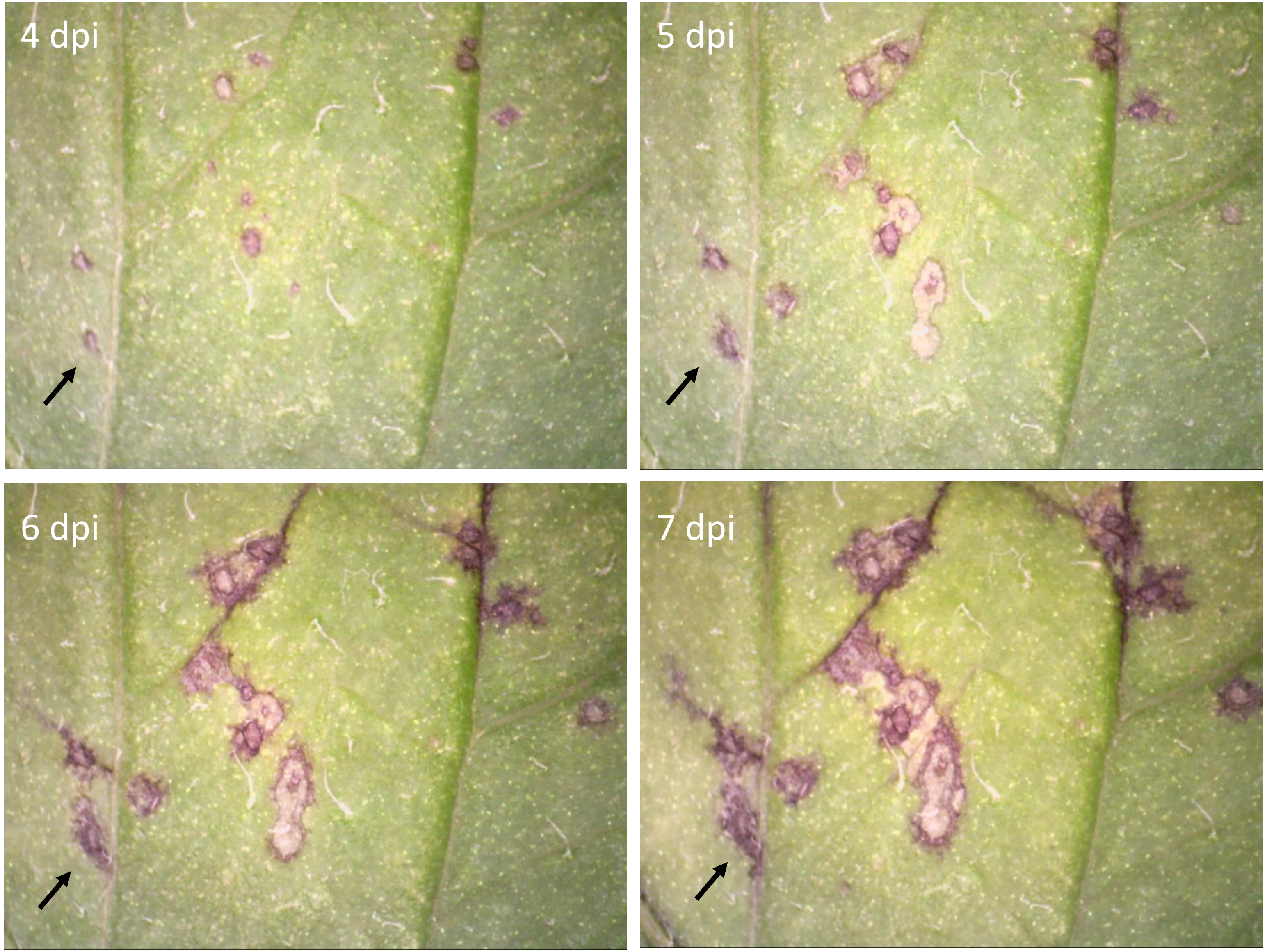

cv. Rywal
Experiment 8
4 dpi – 7 dpi

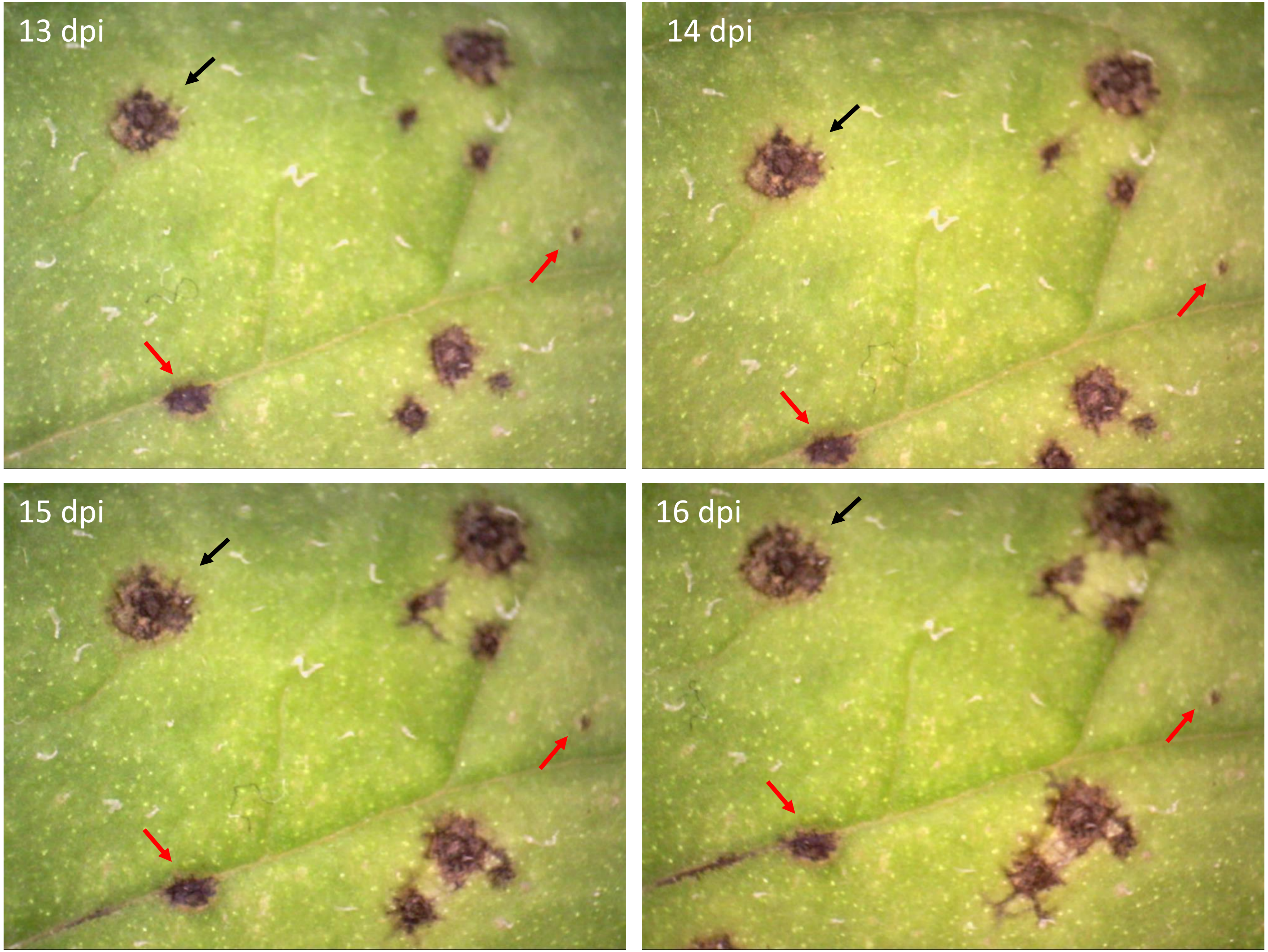

cv. Rywal
Experiment 9
13 dpi – 16 dpi

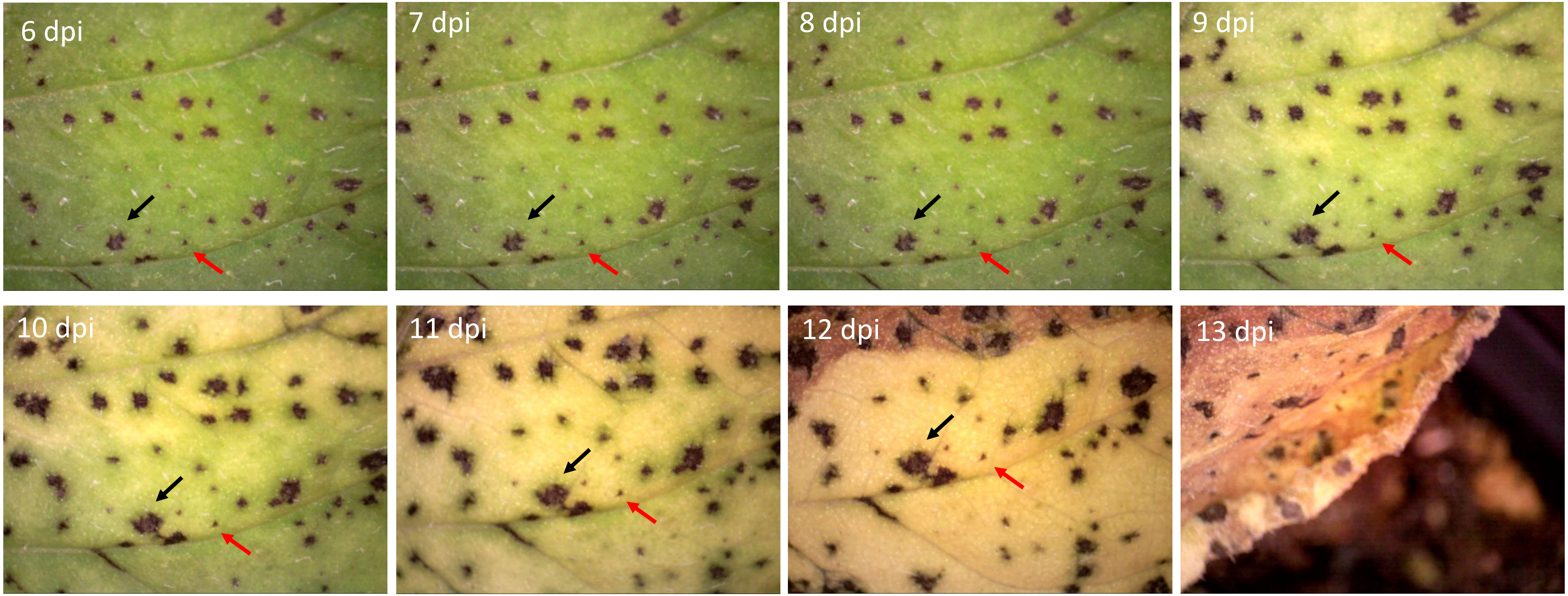

cv. Rywal
Experiment 10
6 dpi – 13 dpi

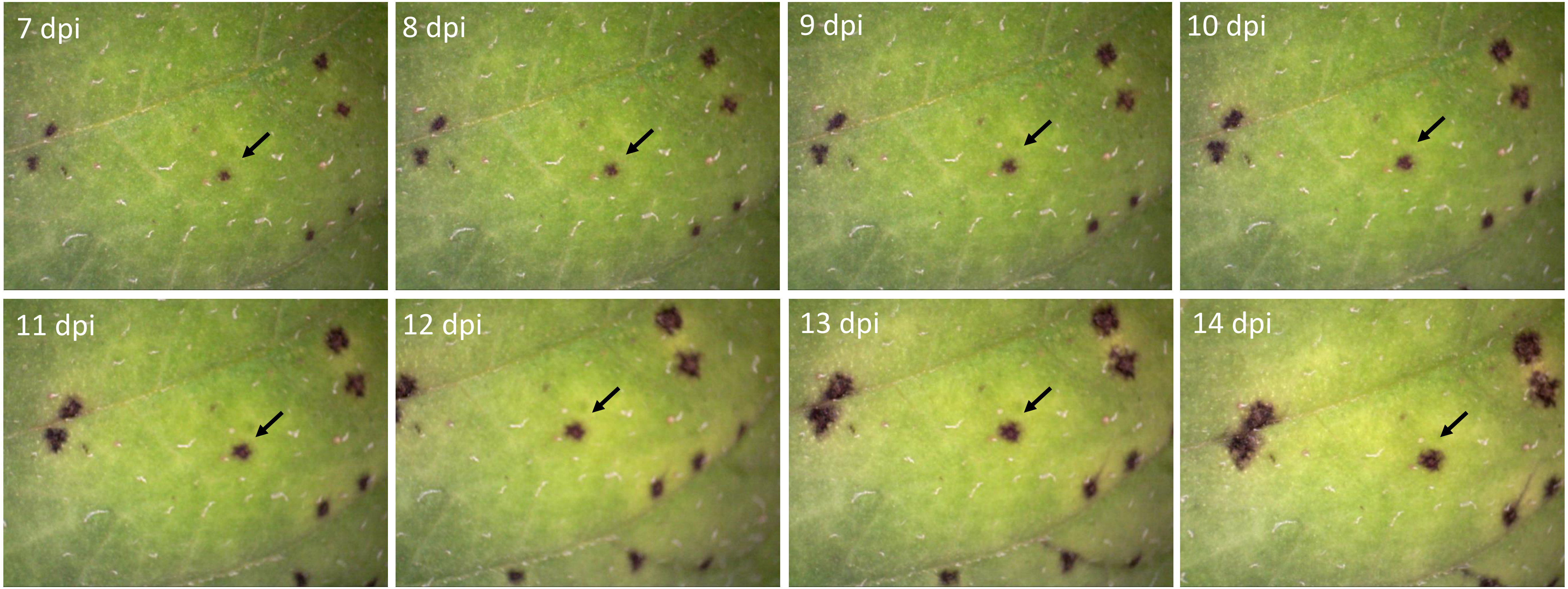

cv. Rywal
Experiment 11
7 dpi – 14 dpi

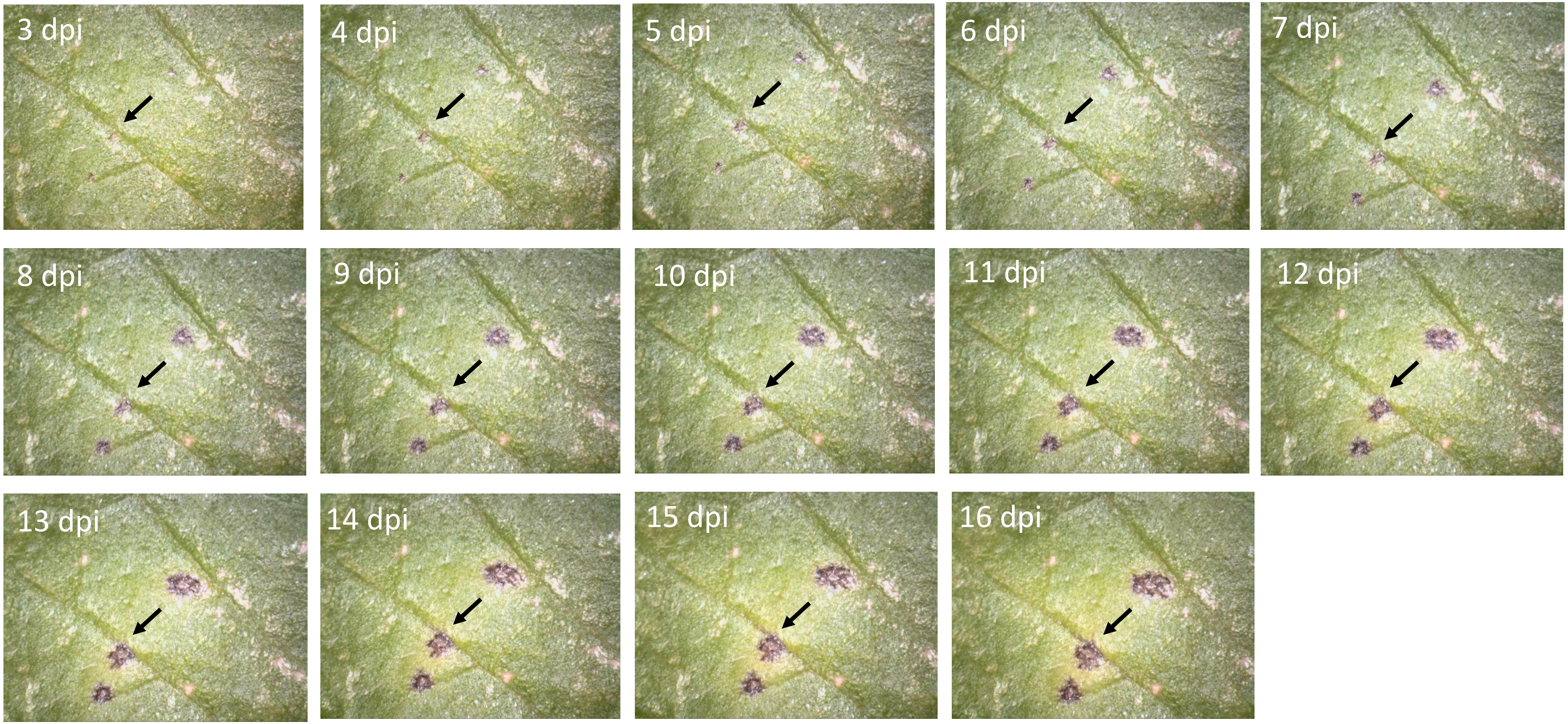

cv. Rywal
Experiment 12
3 dpi – 16 dpi

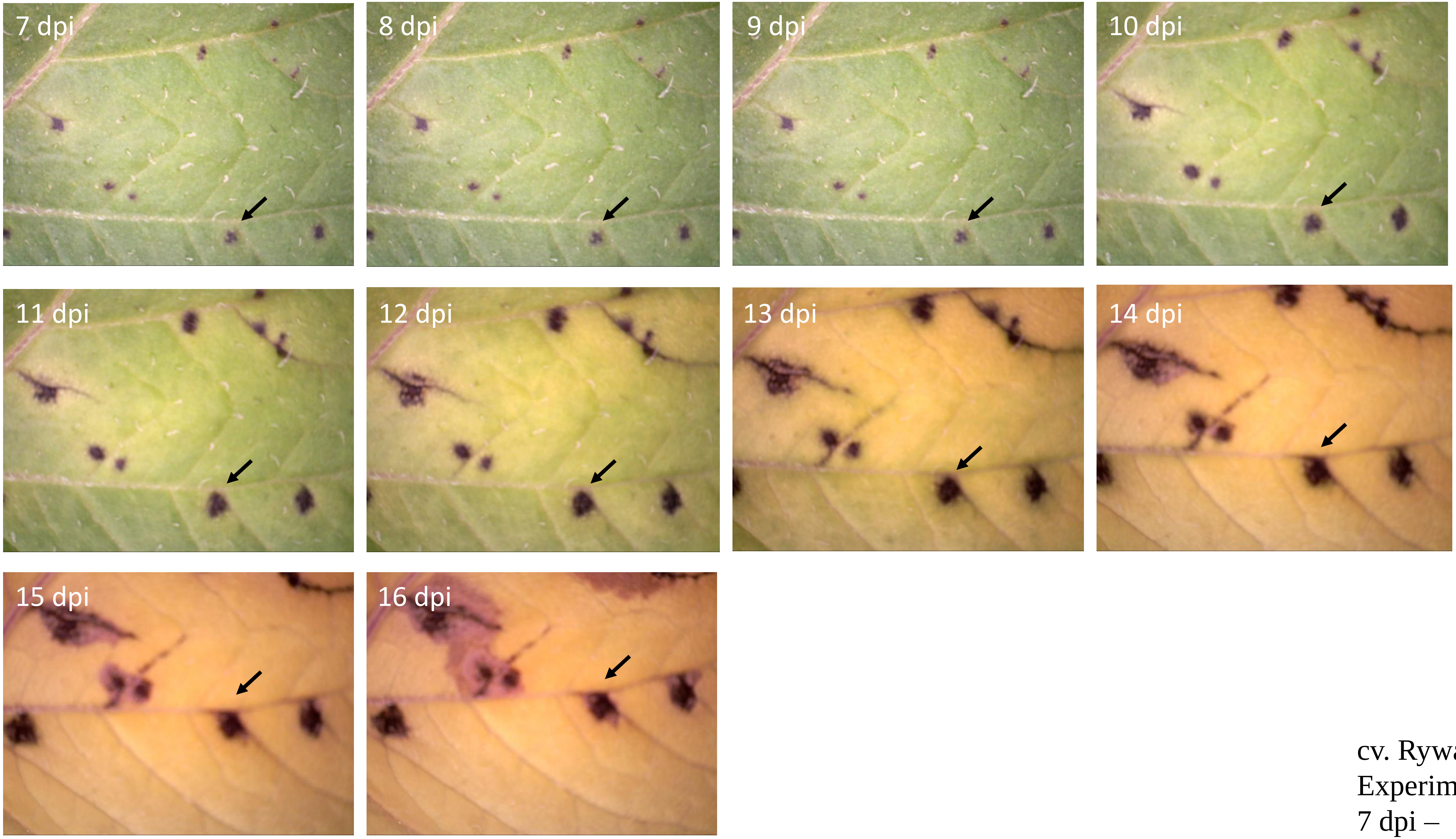

cv. Rywal
Experiment 13
7 dpi – 16 dpi
