# Supplementary material for: Cell Death Is Not Sufficient for the Restriction of Potato Virus Y Spread in Hypersensitive Response-Conferred Resistance in Potato
Source: Front Plant Sci. 2018 Feb 15;9:168. doi: 10.3389/fpls.2018.00168 (PMC5818463; doi:10.3389/fpls.2018.00168)
Supplement: Figure S3 — Number of lesions developed on potato leaves after inoculation with PVY N605-GFP. Lesions were counted in 16 cv. Rywal and 11 NahG-Rywal plants on all three inoculated leaves from 3 to 7 dpi and on 11 dpi to study the dynamics of lesions formation. (A) Numbers of lesions observed on each plant leaf at each time point is shown. X: detached leaf for confocal microscopy, F: the leaf fell off the plant, /: lesions not counted. Some of the leaves (marked with green in the tables) from this experiment were detached from the plants and used to follow virus spread around the lesions (see exp 2 in Supplementary Table 1). (B) Numbers of lesions for each plant leaf from 3 to 11 dpi for selected plants for both genotypes. (C) Standardized imputed cumulative sums of lesions on each leaf from 3 to 11 dpi for selected plants (see Methods for data filtering). [file FigureS3.pdf]

Supplementary Figure 3: Number of lesions developed on potato leaves after inoculation with PVY N605-GFP. Lesions were counted in 16 cv. Rywal and 11 NahG-Rywal plants on all three inoculated leaves from 3 dpi to 7 dpi and on 11 dpi to study the dynamics of lesions formation. (A) Numbers of lesions observed on each plant leaf at each timepoint is shown. X: detached leaf for confocal microscopy, F: the leaf fell off the plant, /: lesions not counted. Some of the leaves (marked with green in the tables) from this experiment were detached from the plants and used to follow virus spread around the lesions (see exp2 in Supplementary Table 1). (B) Numbers of lesions for each plant leaf from 3 to 11 dpi for selected plants for both genotypes. (C) Standardized imputed cumulative sums of lesions on each leaf from 3 to 11 dpi for selected plants (see Methods for data filtering details/description).

(A)

cv. Rywal

**plant no**		**1**			**2**			**3**			**4**			**5**			**6**			**7**			**8**			**9**			**10**			**11**			**12**			**13**			**14**			**15**			**16**	
**dpi/leaf no**	**1**	**2**	**3**	**1**	**2**	**3**	**1**	**2**	**3**	**1**	**2**	**3**	**1**	**2**	**3**	**1**	**2**	**3**	**1**	**2**	**3**	**1**	**2**	**3**	**1**	**2**	**3**	**1**	**2**	**3**	**1**	**2**	**3**	**1**	**2**	**3**	**1**	**2**	**3**	**1**	**2**	**3**	**1**	**2**	**3**	**1**	**2**	**3**
3 dpi	8	2	4	0	2	14	1	0	1	0	1	1	16	22	7	9	3	24	4	1	8	10	0	12	7	10	1	3	0	0	2	1	0	2	1	5	/	0	1	0	4	0	0	0	1	0	0	0
4 dpi	x	x	5	0	2	33	3	0	2	2	1	2	37	37	12	15	11	36	6	3	10	22	1	15	11	22	2	8	0	0	4	7	4	14	8	22	7	2	7	0	6	1	0	1	4	0	2	0
5 dpi	x	x	5	1	2	x	3	0	3	2	2	3	36	46	16	27	x	x	6	4	9	23	1	15	15	22	2	9	1	1	6	5	1	17	12	22	7	1	9	1	9	2	0	1	4	0	4	1
6 dpi	x	x	6	4	2	x	3	0	2	2	3	x	37	x	16	27	x	x	5	4	8	23	1	15	15	22	4	10	1	3	7	10	1	21	14	26	8	1	10	3	13	2	1	3	4	1	4	1
7 dpi	x	x	6	4	2	x	3	0	3	2	3	x	36	x	18	F	x	x	5	x	9	F	1	x	12	23	4	10	1	3	9	10	2	23	13	26	1	1	19	4	15	2	2	6	4	1	4	1
11 dpi	x	x	6	2	2	x	3	2	3	2	2	x	F	x	20	F	x	x	7	x	11	F	2	x	F	x	4	10	1	x	8	F	4	F	11	F	F	1	10	5	14	4	F	7	5	F	3	2

NahG-Rywal

**plant no**		**1**			**2**			**3**			**4**			**5**			**6**			**7**			**8**			**9**			**10**			**11**	
**dpi/leaf no**	**1**	**2**	**3**	**1**	**2**	**3**	**1**	**2**	**3**	**1**	**2**	**3**	**1**	**2**	**3**	**1**	**2**	**3**	**1**	**2**	**3**	**1**	**2**	**3**	**1**	**2**	**3**	**1**	**2**	**3**	**1**	**2**	**3**
3 dpi	1	F	9	0	7	2	7	2	1	16	0	2	5	5	5	2	5	4	0	1	1	2	5	12	2	0	0	0	5	0	2	0	1
4 dpi	5	F	33	7	x	3	30	7	3	58	15	18	11	14	18	10	24	19	2	5	8	9	13	24	22	1	19	2	22	2	31	16	4
5 dpi	5	F	34	13	x	4	29	x	3	64	28	24	15	14	x	12	27	21	2	8	14	11	16	28	25	1	28	3	23	3	35	19	7
6 dpi	5	F	34	12	x	4	28	x	3	x	x	25	15	16	x	11	29	20	2	8	x	12	17	30	21	1	29	3	21	3	35	17	9
7 dpi	5	F	28	11	x	5	21	x	3	x	x	F	17	15	x	12	x	20	2	9	x	10	17	x	18	1	30	4	28	4	37	17	9
11 dpi	F	F	F	F	x	/	F	x	3	x	x	F	F	F	x	F	x	F	F	4	x	F	F	x	x	1	x	2	x	F	F	F	F

(B)

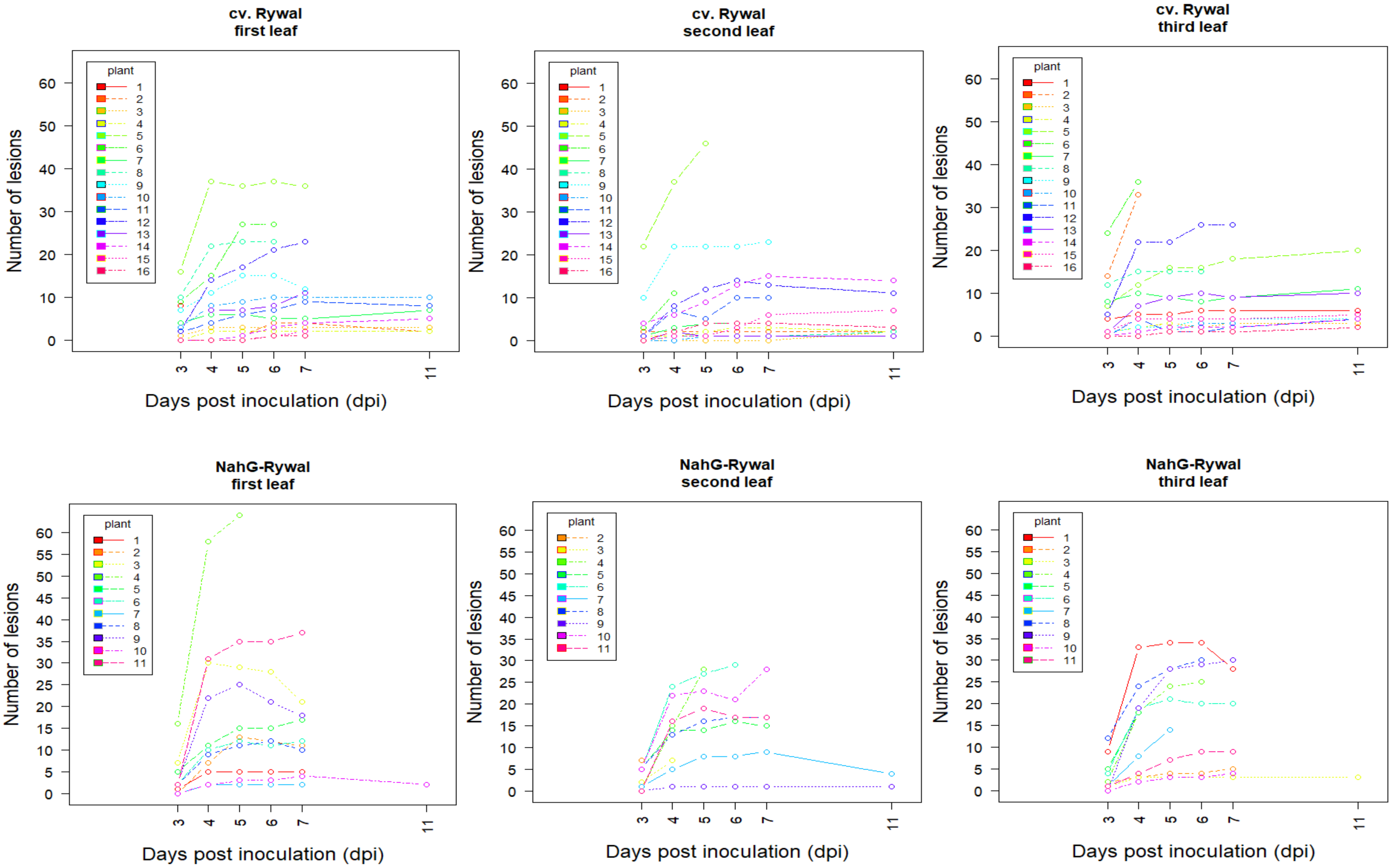

cv. Rywal
first leaf
cv. Rywal
second leaf
cv. Rywal
third leaf
NahG-Rywal
first leaf
NahG-Rywal
second leaf
NahG-Rywal
third leaf
plant
1 2 3 4 5 6 7 8 9 10 11 12 13 14 15 16
2 3 4 5 6 7 8 9 10 11
1 2 3 4 5 6 7 8 9 10 11
Number of lesions
Days post inoculation (dpi)
3 4 5 6 7 11

(C)

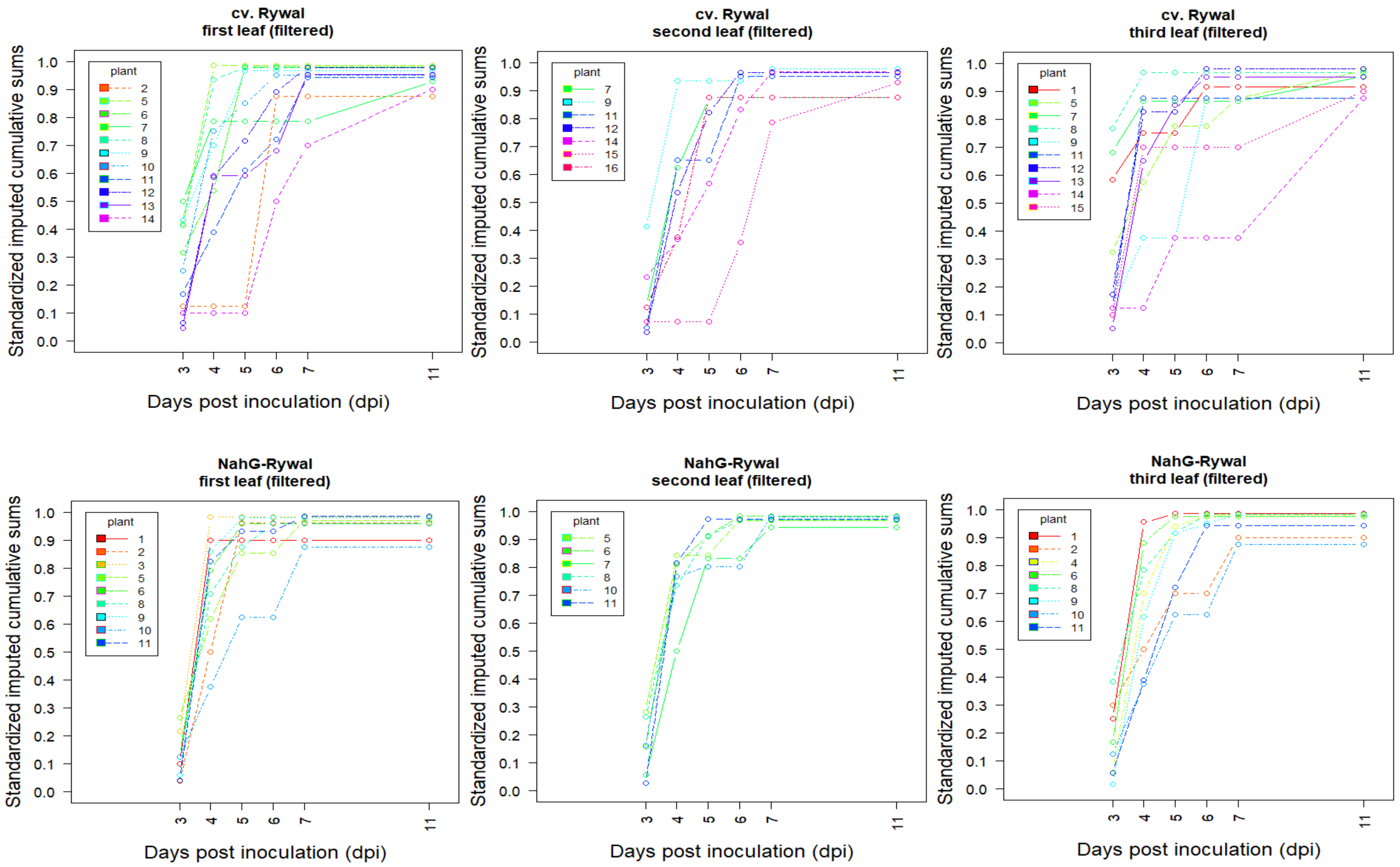
cv. Rywal
first leaf (filtered)
plant
2
5
6
7
8
9
10
11
12
13
14
cv. Rywal
second leaf (filtered)
plant
7
9
11
12
14
15
16
cv. Rywal
third leaf (filtered)
plant
1
5
7
8
9
11
12
13
14
15
NahG-Rywal
first leaf (filtered)
plant
1
2
3
5
6
8
9
10
11
NahG-Rywal
second leaf (filtered)
plant
5
6
7
8
10
11
NahG-Rywal
third leaf (filtered)
plant
1
2
4
6
8
9
10
11
Standardized imputed cumulative sums
0.0
0.1
0.2
0.3
0.4
0.5
0.6
0.7
0.8
0.9
1.0
3
4
5
6
7
11
Days post inoculation (dpi)
