# Supplementary material for: Cell Death Is Not Sufficient for the Restriction of Potato Virus Y Spread in Hypersensitive Response-Conferred Resistance in Potato
Source: Front Plant Sci. 2018 Feb 15;9:168. doi: 10.3389/fpls.2018.00168 (PMC5818463; doi:10.3389/fpls.2018.00168)
Supplement: Supplementary Table 1 — (A) Number of lesions with the PVY N605-GFP accumulation detected outside the cell death zone after inoculation. Results from seven independent experiments (Exp 1–7) are presented for cv. Rywal and NahG-Rywal. At each time point (3–12 dpi) one or two plants (A,B) were analyzed (one leaf per plant). Number of positive lesions (number of lesions with the PVY N605-GFP accumulation detected outside the cell death zone) on the particular day postinoculation/the number of all analyzed lesions are shown. - denotes that a plant was not analyzed at a particular time point. (B) Number of the GFP-containing cells around the positive lesions in cv. Rywal. n.c. denotes that GFP-containing cells around the particular positive lesions were not counted. [file Table1.PDF]

**Supplementary Table 1**: (A) Number of lesions with the PVY N605-GFP accumulation detected outside the cell death zone after inoculation. Results from seven independent experiments (Exp 1-7) are presented for cv. Rywal and NahG-Rywal. At each time point (3-12 dpi) one or two plants (A,B) were analysed (one leaf per plant). Number of positive lesions (number of lesions with the PVY N605-GFP accumulation detected outside the cell death zone) on the particular day post inoculation / the number of all analysed lesions are shown. - denotes that a plant was not analysed at a particular timepoint. (B) Number of the GFP-containing cells around the positive lesions in cv. Rywal. n.c. denotes that GFP-containing cells around the particular positive lesions were not counted.

(A)
**cv. Rywal**

| **Experiment** | **Plant** | **Number of positive lesions at given time point** | | | | | | | |
|---|---|---|---|---|---|---|---|---|---|
| | | **3 dpi** | **4 dpi** | **5 dpi** | **6 dpi** | **7 dpi** | **8 dpi** | **11 dpi** | **12 dpi** |
| Exp1 | Plant A | - | 3/5 | 2/6 | - | 3/8 | 3/10 | - | 2/10 |
| Exp2 | Plant A | 3/4 | 4/9 | 0/3 | 1/4 | 0/10 | - | 1/5 | - |
| | Plant B | - | 2/10 | 0/6 | 1/7 | 0/2 | - | 1/3 | - |
| Exp3 | Plant A | 2/10 | - | - | - | - | - | - | - |
| | Plant B | 4/7 | - | - | - | - | - | - | - |
| Exp4 | Plant A | - | - | 0/24 | 1/15 | 1/20 | 0/18 | - | - |
| | Plant B | - | - | 0/20 | 0/12 | - | - | - | - |
| Exp5 | Plant A | - | - | 1/11 | 3/29 | 3/14 | - | - | - |
| Exp6 | Plant A | 0/10 | 4/9 | 3/10 | 2/9 | 0/11 | - | - | - |
| Exp7 | Plant A | - | - | - | - | - | - | - | - |

**NahG-Rywal**

| **Experiment** | **Plant** | **Number of positive lesions at given time point** | | | | | | | |
|---|---|---|---|---|---|---|---|---|---|
| | | **3 dpi** | **4 dpi** | **5 dpi** | **6 dpi** | **7 dpi** | **8 dpi** | **11 dpi** | **12 dpi** |
| Exp1 | Plant A | - | - | 6/6 | - | 5/5 | - | - | - |
| Exp2 | Plant A | 0/9 | 4/6 | 2/7 | 3/6 | 3/7 | - | 4/5 | - |
| | Plant B | - | 1/2 | 3/4 | 1/4 | 2/2 | - | 2/2 | - |
| Exp3 | Plant A | - | - | - | - | - | - | - | - |
| | Plant B | - | - | - | - | - | - | - | - |
| Exp4 | Plant A | - | - | 3/17 | 4/15 | 2/14 | 2/16 | - | - |
| | Plant B | - | - | 1/11 | - | - | - | - | - |
| Exp5 | Plant A | - | - | 4/9 | 4/10 | - | - | - | - |
| Exp6 | Plant A | 0/10 | 4/10 | 5/10 | 8/10 | 6/8 | - | - | - |
| Exp7 | Plant A | - | - | - | - | - | - | 8/10 | - |

(B)
**cv. Rywal**

| Experiment | Plant | Number of GFP-containing cells around positive lesions | | | | | | | |
|---|---|---|---|---|---|---|---|---|---|
| | | **3 dpi** | **4 dpi** | **5 dpi** | **6 dpi** | **7 dpi** | **8 dpi** | **11 dpi** | **12 dpi** |
| Exp1 | Plant A | - | 3, 3, 2 | 2, 3 | - | 1, 4, >5 | 3, >5, 2 | - | >5, 3 |
| Exp2 | Plant A | >5, 1, 3 | 2, 2, 2, 2 | 0 | 2 | 0 | - | >5 | - |
| | Plant B | - | n.c., 2 | 0 | 1 | 0 | - | >5 | - |
| Exp3 | Plant A | 5, 2 | - | - | - | - | - | - | - |
| | Plant B | >5, >5, 1, 4 | - | - | - | - | - | - | - |
| Exp4 | Plant A | - | - | 0 | 1 | >5 | 0 | - | - |
| | Plant B | - | - | 0 | 0 | - | - | - | - |
| Exp5 | Plant A | - | - | 1 | 3, 1, 5 | 4, 1, >5 | - | - | - |
| Exp6 | Plant A | 0 | 1, 1, 1, 3 | 4, 2, 2 | 3, 1 | 0 | - | - | - |
| Exp7 | Plant A | - | - | - | - | - | - | - | - |
