# Supplementary material for: Cell Death Is Not Sufficient for the Restriction of Potato Virus Y Spread in Hypersensitive Response-Conferred Resistance in Potato
Source: Front Plant Sci. 2018 Feb 15;9:168. doi: 10.3389/fpls.2018.00168 (PMC5818463; doi:10.3389/fpls.2018.00168)
Supplement: Supplementary Table 2 — The probability that the observed lesion was formed until the particular day post inoculation. The probability was calculated after PVY N605-GFP inoculation of cv. Rywal (A) and NahG-Rywal (B) plants by nonlinear regression model with logistic function. [file Table2.PDF]

Supplementary Table 2: The probability that the observed lesion was formed until the particular day post inoculation. The probability was calculated after PVY N605-GFP inoculation of cv. Rywal (A) and NahG-Rywal (B) plants by nonlinear regression model with logistic function.

(A) cv. Rywal

dpi	probability (%)
3	30,86
4	58,56
5	81,73
6	93,40
7	97,82
11	99,98

(B) NahG-Rywal

dpi	probability (%)
3	16,40
4	74,76
5	97,81
6	99,85
7	99,99
11	100,00
