# Supplementary material for: Cell Death Is Not Sufficient for the Restriction of Potato Virus Y Spread in Hypersensitive Response-Conferred Resistance in Potato
Source: Front Plant Sci. 2018 Feb 15;9:168. doi: 10.3389/fpls.2018.00168 (PMC5818463; doi:10.3389/fpls.2018.00168)
Supplement: Supplementary Table 3 — Relative PVY RNA abundance in the systemic leaves in Rywal and NahG-Rywal plants after PVY inoculation. Relative PVY RNA abundance was determined in upper non-inoculated leaves 4 weeks after inoculation with PVY N605-GFP. Results were obtained from two independent experiments. Relative abundance of PVY RNA was followed using quantitative PCR. RNA was isolated from upper non-inoculated leaves using the RN easy Plant Mini Kit (Qiagen) according to the manufacturer's instructions. DNase-treated (0.5 μl DNase per μg RNA; Qiagen) total RNA (1–2 μg) was reverse transcribed using the High Capacity cDNA Reverse Transcription Kit (Applied Biosystems). Amount of virus RNA was normalized to expression of cytochrome oxidase (Cox) according to Baebler et al. (2011). For the detection of Cox and PVY RNA we used TaqMan chemistry as previously described (Baebler et al., 2011). The standard curve method was used for relative quantification using quant Genius (http://quantgenius.nib.si; Baebler et al., 2017). Under LOD: under limit of detection. [file Table3.PDF]

Supplementary Table 3: Relative PVY RNA abundance in the systemic leaves in Rywal and NahG-Rywal plants after PVY inoculation. Relative PVY RNA abundance was determined in upper non-inoculated leaves 4 weeks after inoculation with PVY N605-GFP. Results were obtained from two independent experiments. Relative abundance of PVY RNA was followed using quantitative PCR. RNA was isolated from upper non-inoculated leaves using the RNeasy Plant Mini Kit (Qiagen) according to the manufacturer's instructions. DNase-treated (0.5 µl DNase per µg RNA; Qiagen) total RNA (1–2 µg) was reverse transcribed using the High Capacity cDNA Reverse Transcription Kit (Applied Biosystems). Amount of virus RNA was normalized to expression of cytochrome oxidase (Cox) according to Baebler et al., 2011. For the detection of Cox and PVY RNA we used TaqMan chemistry as previously described (Baebler et al., 2011). The standard curve method was used for relative quantification using quantGenius (http://quantgenius.nib.si; Baebler et al., 2017). Under LOD: under limit of detection.

Experiment	Genotype	Plant No.	Leaf No.	Relative viral RNA abundance
Exp 1	Rywal	P1	L1	Under LOD
		P2	L1	Under LOD
Exp 2		P3	L1	Under LOD
			L2	Under LOD
		P4	L1	Under LOD
			L2	Under LOD
Exp 1	NahG-Rywal	P1	L1	3,8
		P2	L1	9,3
Exp 2		P3	L1	1,0
			L2	1,8
		P4	L1	5,6
			L2	1,7
